# Heck Transformations of Biological Compounds Catalyzed by Phosphine-Free Palladium

**DOI:** 10.3390/molecules23092227

**Published:** 2018-09-01

**Authors:** Stanisława Tarnowicz-Ligus, Anna M. Trzeciak

**Affiliations:** Faculty of Chemistry, University of Wrocław, 14 F. Joliot-Curie, 50-383 Wrocław, Poland; stanislawa.tarnowicz@chem.uni.wroc.pl

**Keywords:** palladium, arylation, Heck coupling, cinnamyl alcohol, linalool, eugenol, estragole

## Abstract

The development and optimization of synthetic methods leading to functionalized biologically active compounds is described. Two alternative pathways based on Heck-type reactions, employing iodobenzene or phenylboronic acid, were elaborated for the arylation of eugenol and estragole. Cinnamyl alcohol was efficiently transformed to saturated arylated aldehydes in reaction with iodobenzene using the tandem arylation/isomerization sequential process. The arylation of cinnamyl alcohol with phenylboronic acid mainly gave unsaturated alcohol, while the yield of saturated aldehyde was much lower. Catalytic reactions were carried out using simple, phosphine-free palladium precursors and water as a cosolvent, following green chemistry rules as much as possible.

## 1. Introduction

The Heck reaction is one of the most convenient methods for C–C bond formation applied in the preparation of many molecules developed as pharmaceuticals, agrochemicals, and functional materials [1,2,3,4,5]. In particular, it can be applied for allyl alcohol arylation catalyzed by Pd(0) nanoparticles [6]. On the other hand, Heck arylation suffers some drawbacks, such as the application of expensive and toxic aryl halides and the generation of harmful HX waste during the process [7,8]. The halide-free Heck-type reaction, employing arylboronic acid as an aryl source, can be considered an environmentally benign alternative [9,10,11,12]. The direct heteroarylation procedure presents another green pathway leading to arylated organic products [13,14].

Cinnamyl alcohol, eugenol, and estragole are natural compounds. The arylation of these compounds has so far not been intensively developed. Cinnamyl alcohol (3-phenyl-2-propen-1-ol) is used to obtain many cosmetics, perfumes, and detergents [15]. This alcohol has been arylated with iodobenzene to produce α,β-unsatured alcohols by using TBAB (tetrabutylammonium bromide) and NaHCO_3_ as bases [16]. In analogous reactions performed in the presence of TBAA or without a base, exclusively carbonyl products have been formed [16]. The arylation of cinnamyl alcohol with different aryldiazonium salts under mild conditions in the presence of a Pd_2_(dba)_3_ catalyst has been reported [17]. The regio- and stereoselective formation of (Z)-2,3-diarylallylic alcohols has been carried out using a palladium catalyst and the n-Bu_4_NOAc base in toluene [18]. Arylboronic acid has been used for the arylation of cinnamic phenyl ether in the presence of Pd(OAc)_2_ and a hydrazone ligand [19].

Eugenol (1-allyl-3-methoxy-4-hydroxybenzene) is extracted from clove oil and marjoram, and it is present in spices such as basil, cinnamon, and nutmeg [20]. Eugenol can be employed in the synthesis of natural products, bioactive compounds, heterocycles, macrocycles, and polymers [21]. A methyl eugenol derivative has been arylated according to the Heck and oxidative Heck procedures to give products showing inhibitory effects on breast cancer cell metathesis [22]. Estragole (*p*-allylanisole) occurs in basil oil, and it has antioxidative, antimicrobial [23], and anesthetic [24] properties. The arylation of estragole has been performed with bromobenzene and palladium polyether diphosphinite complex anchored in polyethylene glycol [25]. Linalool (3,7-dimethyl-1,6-octadien-3-ol) is a tertiary monoterpene alcohol which is a major component of the *Coriandrum sativum* essential oil. Moreover, it has very wide applications in the synthesis of fine fragrances, cosmetics, detergents, and other commercial derivatives [26,27,28]. Linalool leads to the corresponding (*E*)-1-arylalk-1-en-3-ol under the Heck conditions with a Pd-Tedicyp catalyst (Tedicyp = *cis*, *cis*, *cis*, -1,2,3,4-tetrakis(diphenylphosphinomethyl)cyclopentane) [29,30].

Considering a wide spectrum of biological properties of the above-mentioned compounds, interesting activities of their aryl derivatives can also be expected, as has already been shown for the eugenol derivative [22]. In our work, we focused on the application of phosphine-free palladium precursors (Pd(OAc)_2_, PdCl_2_cod, or PdCl_2_(CH_3_CN)_2_) in two Heck-type arylation reactions employing iodobenzene or phenylboronic acid as an arylating agent. The application of phenylboronic acid avoids the formation of halide waste, in agreement with green chemistry rules. We also tested using water as a cosolvent in the reactions studied.

## 2. Results and Discussion

**Scheme 1:**
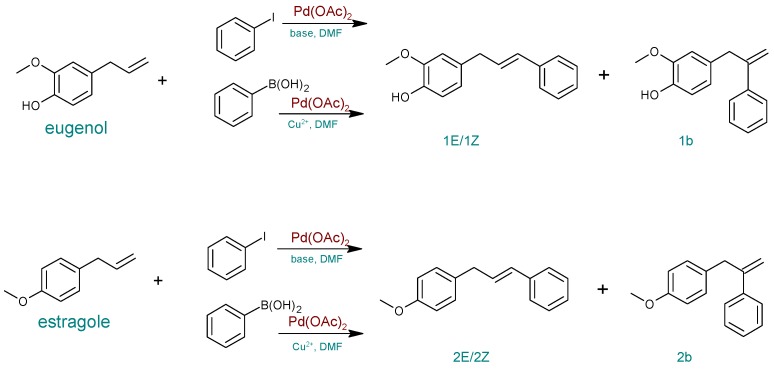
Heck and Heck-type arylation of eugenol or estragole.

In order to get arylated derivatives of eugenol and estragole, two methods were tested and optimized. The first one was the Heck coupling with iodobenzene. In the second procedure, based on the Heck-type reaction, phenylboronic acid was employed as an arylating agent (Scheme 1). In this method, the base was eliminated from the system; however, the addition of a Cu(II) salt as an oxidant regenerating the catalytic amount of Pd(II) from Pd(0) was necessary [10,11,12,23]. The Heck-type reaction with the application of phenylboronic acid limits the formation of halide waste materials.

Table 1 presents the results of the Heck arylation of eugenole with iodobenzene. Three products, **1E**, **1Z**, and **1b**, were identified, and the amount of **1b** was below 3% in all cases, while **1E** was the main product. The first experiments, carried out for 3 h at 100 °C in DMF as a solvent, resulted in a 100% conversion of PhI with the formation of 81% of **1E** and 16% of **1Z**. Very similar results were obtained after 1 h (95% conversion). Further studies showed that the Heck arylation can be successfully performed in a DMF:water mixture (DMF:water = 4:1 or 2.5:2.5). The reaction rate slowed down at a higher content of water, and the yield of **1E** was only 69% after 1 h. However, after 2 h, the conversion of PhI achieved 100% with a 79% yield of **1E**. The conversion of PhI depended on the reaction temperature: it decreased at lower temperatures, 80 °C and 60 °C, to 90% and 74%, respectively, after 3 h (Table 1, entries 1, 11 and 12). On the other hand, product **1b** was not formed at lower temperatures. Considering the effect of the catalyst type, PdCl_2_(CH_3_CN)_2_ turned out to be weaker than Pd(OAc)_2_, resulting in a 16% lower conversion (Table 1, entry 2 and 9).

Next, the Heck arylation of estragole with iodobenzene was studied (Table 2). After 3 h of a reaction carried out at 100 °C, the conversion of iodobenzene reached 96%, and the yields of the main products, **2E** and **2Z**, were comparable, 47% and 42%, respectively. The presence of water resulted in a decrease in PhI conversion to 61% at the DMF:H_2_O ratio equal 4:1. In all experiments, the amount of product **2b** was as low as 3–7%.

In the alternative Heck-type procedure with phenylboronic acid used instead of iodobenzene, the base was eliminated and copper salts, Cu(OAc)_2_ or Cu(OAc)_2_.·H_2_O, were added (Scheme 1, Table 3). The best result, 92% conversion of eugenol, was achieved after 4 h at 100 °C (Table 3, entry 8). Two products, **1E** and **1Z**, were formed in comparable amounts, 46 and 39%, respectively. In almost all experiments performed according to this procedure, the yields of **1E** and **1Z** were similar. This is different from the Heck method with iodobenzene, which favored product **1E** over **1Z**. Only in one case, in a shorter reaction of 3 h, was the excess of **1E** over **1Z** significant (26%) (Table 3, entry 7). However, the prolongation of this reaction to 4 h resulted in an increase in the yield of **1E**. The Heck-type procedure with phenylboronic acid was successful only when copper salts were employed as palladium oxidants. In contrast, arylation products were not formed in reactions performed in air (Table 3).

Eugenol reacted faster according to the Heck procedure, and the conversion of PhI was 100% already after 1 h (Table 1, entry 5). In the Heck-type reaction, a conversion of 92% was achieved in 4 h (Table 3, entry 8).

Estragole was efficiently arylated with phenylboronic acid under the same conditions (Table 4). In this case, the positive effect of copper salts on the reaction course was also noted, while products were not formed in air atmosphere without copper. The best result, 92% conversion of estragole, was obtained after 4 h at 100 °C. Both products, **2E** and **2Z**, were formed in comparable amounts, 47 and 41%, respectively. In general, this method gave similar results to that with iodobenzene.

**Scheme 2:**
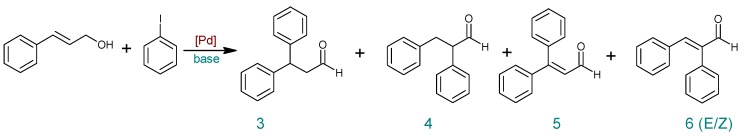
Heck coupling of cinnamyl alcohol.

The arylation of cinnamyl alcohol with iodobenzene produced a mixture of products with saturated aldehydes **3** and **4** being the main ones (Scheme 2). The formation of these products can be explained by a tandem process involving the isomerization of the Heck-reaction product, namely di-arylated allylic alcohol, produced in the first step [16]. The small amounts of unsaturated aldehydes **5** and **6** were probably formed by the Pd(II)-catalyzed dehydrogenation of **3** and **4** [31].

The data collected in Table 5 indicate the positive effect of water on the reaction course. For example, the yield of product **3** was 13%, and that of product **4** was 29%, after a 6 h reaction in DMF. When the same reaction was carried out in DMF:H_2_O (1:1), the yield of products **3** and **4** increased to 34 and 57%, respectively. Moreover, in the presence of water, the formation of a biphenyl side product was completely suppressed (Table 5, entry 3). Unfortunately, attempts to increase the amount of water to the DMF:H_2_O ratio of 1:4 resulted in a biphenyl yield increase to 19%, although the conversion of iodobenzene was quite high (93%) (Table 5, entry 4). Good results were also obtained in the presence of a TBAB additive (Table 5, entry 2). The formation of side products **5** and **6** was noted in few cases.

**Scheme 3:**
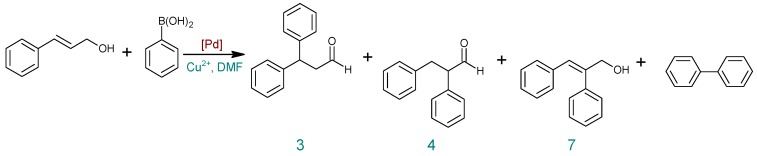
The Heck-type arylation of cinnamyl alcohol with phenylboronic acid.

Next, the reaction of cinnamyl alcohol with phenylboronic acid was studied (Scheme 3). In these conditions, PdCl_2_cod was not active, and Pd(OAc)_2_ and Pd_2_(dba)_3_ were used as catalysts. Interestingly, the unsaturated alcohol **7**, which was not obtained in reaction with iodobenzene, was formed as the main product in this case (Table 6). Arylation with phenylboronic acid was rather fast, and, already after 1 h at 50 °C, 100% of the alcohol was converted to afford 74% of **7** together with 20% and 6% of products **3** and **4**, respectively. At a higher temperature, 100 °C, the yield of the side product, biphenyl, increased.

Considering the activity of the catalysts used, it is worth noting that the activity of the Pd(0) complex was similar to that of the Pd(II) compound. The conversion of the substrate was high for both catalysts; however, Pd_2_(dba)_3_ preferably produced product **7**, due to slow isomerization to **4.** According to the well-accepted mechanism of the oxidative Heck coupling, Pd(II) is assumed to be catalytically active [10,11,23]. However, we have no evidence of the oxidation of Pd_2_(dba)_3_ in our system.

**Scheme 4:**
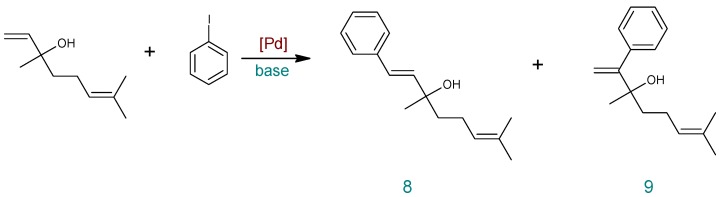
Heck coupling of linalool.

The last substrate studied was linalool (Scheme 4). It reacted smoothly with iodobenzene forming products **8** and **9**.

As shown in Table 6, the conversion of iodobenzene after 3 h was 42–47%, while it increased significantly, to 61–95%, after a change of the solvent from DMF alone to a DMF/water mixture (Table 7, entries 2 and 4). Considering the base, NaOAc was found the most efficient, enabling the formation of 66% of product **8** and 29% of product **9**. Unfortunately, linalool did not react with phenylboronic acid under Heck-type conditions with Pd(OAc)_2_ as a catalyst.

## 3. Materials and Methods

### 3.1. Reactants

Iodobenzene, eugenol, estragole, cinnamyl alcohol, linalool, and phenylboronic acid (Sigma Aldrich, Poznań, Poland) were used without purification or drying.

### 3.2. General Procedures

#### 3.2.1. Heck Reaction (Method A)

The reaction was carried out in a 50 cm^3^ Schlenk tube. The solid substrates, the base (2 mmol), and the Pd catalyst (1 × 10^−5^ mol) were weighed and placed in the Schlenk tube under an N_2_ atmosphere. Next, olefin (1 mmol), iodobenzene (1 mmol), and 5 cm^3^ of the solvent (DMF alone or DMF mixed with water) were added with a pipette. The Schlenk tube was closed with a rubber plug, and the reaction mixture was stirred at 60–100 °C in oil bath. After the given reaction time, the Schlenk tube was cooled down, and the organic products were separated by extraction with diethyl ether (three times with 5, 4, and 2 cm^3^). For better phase separation, 5 cm^3^ of water was added, and the products were GC–MS analyzed (Hewlett Packard 8454A (Palo Alto, CA, USA)) with mesitylene (0.1 cm^3^) as the internal standard. The GC yield given in the Table means the substrate conversion to a given product. Physicochemical data of reaction products can be found in Supplementary Materials. 

#### 3.2.2. Heck-Type Reaction (Method B)

The reaction was carried out in a 50 cm^3^ Schlenk tube. The solid substrates, copper (II) acetate (2 mmol), phenylboronic acid (1.5 mmol), and the Pd catalyst (1 × 10^−5^ mol) were weighed and placed in the Schlenk tube under an N_2_ atmosphere. Next, olefin (1 mmol) and 5 cm^3^ of the solvent (DMF alone or DMF mixed with water) were added with a pipette. The Schlenk tube was closed with a rubber plug, and the reaction mixture was stirred at 50–120 °C in oil bath. After the given reaction time, the Schlenk tube was cooled down, and the organic products were separated by extraction with diethyl ether (three times with 5, 4, and 2 cm^3^). For better phase separation, 5 cm^3^ of water was added, and the products were GC–MS analyzed (Hewlett Packard 8454A) with mesitylene (0.1 cm^3^) as the internal standard. The GC yield given in the Table means the substrate conversion to a given product. Physicochemical data of reaction products can be found in Supplementary Materials. 

#### 3.2.3. Heck-Type Reaction (Method C)

The reaction was carried out in a 50 cm^3^ Schlenk tube. The solid substrates, the base (2 mmol), and the PdCl_2_cod (1 × 10^−5^ mol) were weighed and placed in the Schlenk tube under an N_2_ atmosphere. Next, iodobenzene (1 mmol), cinnamyl alcohol or linalool (1 mmol), and 5 cm^3^ of the solvent (DMF alone or DMF mixed with water) were added with a pipette. The Schlenk tube was closed with a rubber plug, and the reaction mixture was stirred at 50–100 °C in oil bath. After the given reaction time, the Schlenk tube was cooled down, and the organic products were separated by extraction with diethyl ether (three times with 7, 3, and 2 cm^3^). For better phase separation, 5 cm^3^ of water was added, and the products were GC–MS analyzed (Hewlett Packard 8454A) with mesitylene (0.1 cm^3^) as the internal standard. The GC yield given in the Table means the substrate conversion to a given product. Physicochemical data of reaction products can be found in Supplementary Materials. 

## 4. Conclusions

We presented two efficient methods based on palladium-catalyzed Heck procedures, leading to arylated products of biological importance. In particular, it was discovered that water serving as a co-solvent significantly enhanced product yield.

The Heck-type reaction employing phenylboronic acid and Cu^2+^ salts was tested for the first time in the arylation of eugenol and estragole, and the obtained results were very promising. In this procedure, the iodobenzene substrate was substituted by phenylboronic acid, which improved the economic and environmental aspects. The same method applied to cinnamyl alcohol enabled the preparation of arylated unsaturated alcohol in 1 h at 50 °C.

In the Heck coupling of cinnamyl alcohol with iodobenzene, diarylated saturated aldehydes were obtained. Also in this case, the addition of water resulted in an increase in iodobenzene conversion by over 20%, and 57% of 2,3-diphenylpropanal was formed. Similarly, the arylation of linalool in a DMF:H_2_O mixture gave better results (66% of product **8** and 29% of product **9**) than a reaction carried out in the absence of water (33% of product **8** and 14% of product **9**).

## Figures and Tables

**Table 1:** Results of the Heck arylation of eugenol with iodobenzene.

Entry	Catalyst	Base	DMF:H_2_O	T (°C)	Time (h)	PhI Conversion (%)	Yield (%) ^a^		
							1E	1Z	1b
1	Pd(OAc)_2_	K_2_CO_3_	DMF	100	3	100	81	16	3
2			DMF	100	1	95	78	15	2
3			4:1	100	3	100	84	14	2
4			4:1	100	2	100	85	15	0
5			4:1	100	1	100	85	13	1
6			2.5:2.5	100	2	100	79	21	0
7			2.5:2.5	100	1	93	69	23	1
9	PdCl_2_(CH_3_CN)_2_		DMF	100	1	79	74	5	0
10	Pd(OAc)_2_		4:1	100	40 min	74	65	7	2
11			4:1	80	3	90	80	10	0
12			4:1	60	3	74	68	6	0

Reaction conditions (method A): PhI (1 mmol), eugenol (1 mmol), K_2_CO_3_ (2 mmol), [Pd] (1 × 10^−5^ mol), DMF or mixture of DMF with H_2_O (5 cm^3^). ^a^ GC yield.

**Table 2:** Results of the Heck arylation of estragole with iodobenzene.

Entry	Catalyst	Base	DMF:H_2_O	T (°C)	Time (h)	PhI Conversion (%)	Yield (%) ^a^		
							2E	2Z	2b
1	Pd(OAc)_2_	K_2_CO_3_	DMF	80	3	71	36	30	5
2			DMF	100	3	96	47	42	7
4			DMF	100	1	77	40	31	6
5			2.5:2.5	100	1	41	24	14	3
3			4:1	100	3	61	35	22	4
6			4:1	100	1	15	11	4	0
7			4:1	80	3	37	28	9	0

Reaction conditions (method A): PhI (1 mmol), estragole (1 mmol), K_2_CO_3_ (2 mmol), [Pd] (1 × 10^−5^ mol), DMF or a mixture of DMF and H_2_O (5 cm^3^). ^a^ GC yield.

**Table 3:** Results of the Heck-type arylation of eugenol with phenylboronic acid.

Entry	Catalyst	Base	Modification	T (°C)	Time (h)	Eugenol Conversion (%)	Yield (%) ^a^		
							1E	1Z	1b
1	Pd(OAc)_2_	KOH	Air	50	3	0	0	0	0
2		Na_2_CO_3_	Air	50	3	0	0	0	0
3		K_2_CO_3_	Air	50	3	0	0	0	0
3	PdCl_2_cod	˗	Cu(OAc)_2_	120	4	55	21	21	7
4		˗		100	4	25	9	6	10
5		˗	Cu(OAc)_2_.·H_2_O	100	4	53	26	17	9
6		˗	Cu(OAc)_2_	120	4	57	26	25	6
7	Pd(OAc)_2_	˗		100	3	81	51	26	4
8		˗		100	4	92	46	39	4

Reaction conditions (method B): eugenol (1 mmol), phenylboronic acid (1.5 mmol), base (2 mmol), Cu^2+^ salt (2 mmol), [Pd] (1 × 10^−5^ mol), DMF (5 cm^3^). ^a^ GC yield.

**Table 4:** Results of the Heck-type reaction of estragole with phenylboronic acid.

Entry	Catalyst	Base	Modification	T (°C)	Time (h)	Estragole Conversion (%)	Yield (%) ^a^		
							2E	2Z	2b
1	Pd(OAc)_2_	KOH	Air	50	3	0	0	0	0
2		Na_2_CO_3_	Air	50	3	0	0	0	0
3	PdCl_2_cod	˗	Cu(OAc)_2_	120	4	64	34	28	2
4		˗		100	4	34	22	12	0
5		˗	Cu(OAc)_2_.·H_2_O	100	4	59	32	24	3
6	Pd(OAc)_2_	˗	Cu(OAc)_2_	100	2.5	89	46	39	4
7		˗		100	3	90	49	37	4
8		˗		100	4	92	47	41	4

Reaction conditions (method B): estragole (1 mmol), phenylboronic acid (1.5 mmol), base (2 mmol), Cu^2+^ salt (2 mmol), [Pd] (1 × 10^−5^ mol), DMF (5 cm^3^). ^a^ GC yield.

**Table 5:** Results of the Heck reaction of cinnamyl alcohol with iodobenzene.

Entry	Catalyst	Base	Modification	Time (h)	PhI Conversion (%)	Yield (%) ^b^				
						Bif.	3	4	5	6
1	PdCl_2_cod	NaOAc	˗	6	68	15	13	29	7	2
2	PdCl_2_cod		TBAB ^a^	6	91	5	28	52	3	3
3	PdCl_2_cod		DMF:H_2_O (1:1)	6	93	˗	34	57	˗	˗
4	PdCl_2_cod		DMF:H_2_O (4:1)	6	93	19	14	53	˗	˗
5	PdCl_2_cod	K_2_HPO_4_	˗	6	30	˗	11	19	˗	˗
6	PdCl_2_cod		DMF:H_2_O (1:1)	6	56	10	18	28	˗	˗
7				24	96	2	8	72	˗	11
8	PdCl_2_cod		DMF:H_2_O (1:4)	6	68	10	13	40	˗	˗
9	PdCl_2_cod	Et_3_N	˗	6	68	24	22	18	˗	˗
10			DMF:H_2_O (1:1)	6	67	5	26	36	˗	˗

Reaction conditions (method C): PhI (1 mmol), cinnamyl alcohol (1 mmol), base (2 mmol), [Pd] (1 × 10^−5^ mol), DMF or DMF mixed with H_2_O (5 cm^3^), 100 °C; ^a^ 1 g of TBAB was added. ^b^ GC yield.

**Table 6:** Results of the Heck-type arylation of cinnamyl alcohol with phenylboronic acid.

Entry	Catalyst	Salt Cu^2+^	T (°C)	Time (h)	Cinnamyl Alcohol Conversion (%)	Yield (%) ^b^		
						3	4	7
1	Pd(OAc)_2_	Air	100	4	0	0	0	0
2		Cu(OAc)_2_	100	6	100	37	14	49
3			100	4	100	40	16	44
4			50	1	90	18	6	63
5	Pd_2_(dba)_3_		50	4	100	19	6	75
6			50	1	100	20	6	74
7 ^a^			50	1	63	13	5	45
8			50	0.5	90	25	8	56

Reaction conditions (method B): cinnamyl alcohol (1 mmol), phenylboronic acid (1.5 mmol), Cu^2+^ salt (2 mmol), [Pd] (1 × 10^−5^ mol), DMF (5 cm^3^), ^a^ [Pd] (0.5 × 10^−5^ mol). ^b^ GC yield.

**Table 7:** Results of the Heck arylation of linalool with iodobenzene.

Entry	Catalyst	Base	Modification	Time (h)	PhI Conversion (%)	Yield (%) ^a^	
						8	9
1	PdCl_2_cod	NaOAc	˗	3	47	33	14
2			DMF:H_2_O (4:1)	3	95	66	29
3		K_2_HPO_4_	˗	3	42	21	21
4			DMF:H_2_O (4:1)	3	65	48	17
5		Et_3_N	˗	3	42	29	12
6			DMF:H_2_O (4:1)	3	61	45	12

Reaction conditions (method C): PhI (1 mmol), linalool (1 mmol), base (2 mmol), [Pd] (1 × 10^−5^ mol), 100 °C, DMF or mixed DMF with H_2_O (5 cm^3^). ^a^ GC yield.

## Supplementary Materials

Supplementary materials are available on line.
