# Supplementary material for: Heck Transformations of Biological Compounds Catalyzed by Phosphine-Free Palladium
Source: Molecules. 2018 Sep 1;23(9):2227. doi: 10.3390/molecules23092227 (PMC6225119; doi:10.3390/molecules23092227)
Supplement: Supplementary file 1 [file molecules-23-02227-s001.pdf]

Phosphine-Free Palladium Catalyzed Heck reaction with using natural compounds

Stanisława Tarnowicz-Ligus, Anna M. Trzeciak*

Table of Contents

**1**	**General Remarks**	**S2**
**2**	**Control experiments and date of products**	**S2-S22**

General Remarks: Products have been characterized by the GC-MS (HP II 5890 + Mass Selective Detector HP 5971A) and by ^{1}H NMR and ^{13}C NMR. NMR spectra have been measured for the post-reaction mixtures. Unless noted, ^{1}H NMR spectra were recorded on Bruker 500 MHz in $CDCl_3$, ^{13}C NMR spectra were recorded on 125 MHz in $CDCl_3$.

Control experiment 1:

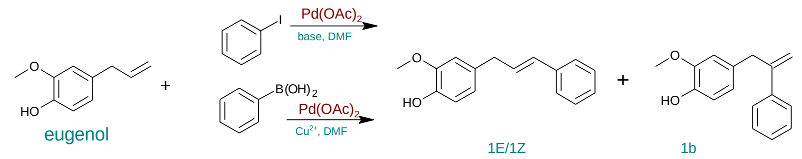

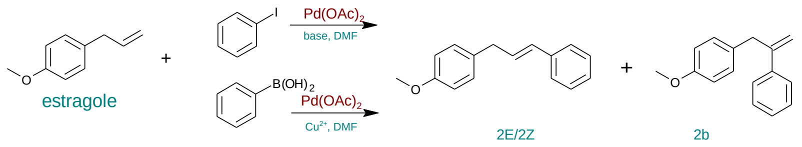

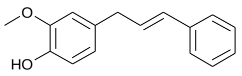

1E/1Z (2-methoxy-4-(3-phenyl-2-propen-1-yl)-phenol)

(**1E/1Z**) MS: m/z (%) = 91 (30), 115 (37), 131 (16), 165 (12), 179 (17), 207 (23), 240 (100) [M^{+}]

^{1}H–NMR (500 MHz, $CDCl_3$): δ = 7.37–7.33 (m, 2,6H, H_{Ar}), 7.31–7.27 (m, 2,4H, H_{Ar}), 6.91 (d, *J* = 12.4Hz, 3H, H_{Ar}), 6.42 (d, *J* = 15.8Hz, 1H, CH), 6.28–6.22 (m, 1H, CH), 3.86 (s, 3H, CH_3), 3.56 (d, *J* = 7.1Hz, 2H,CH_2) ppm.

^{13}C–NMR (125 MHz, $CDCl_3$): δ = 146.82, 145.28, 140.50, 130.89, 130.12, 128.75, 128.54, 126.97, 126.20, 119.82, 114.59, 108.27, 55.90, 39.36 ppm.

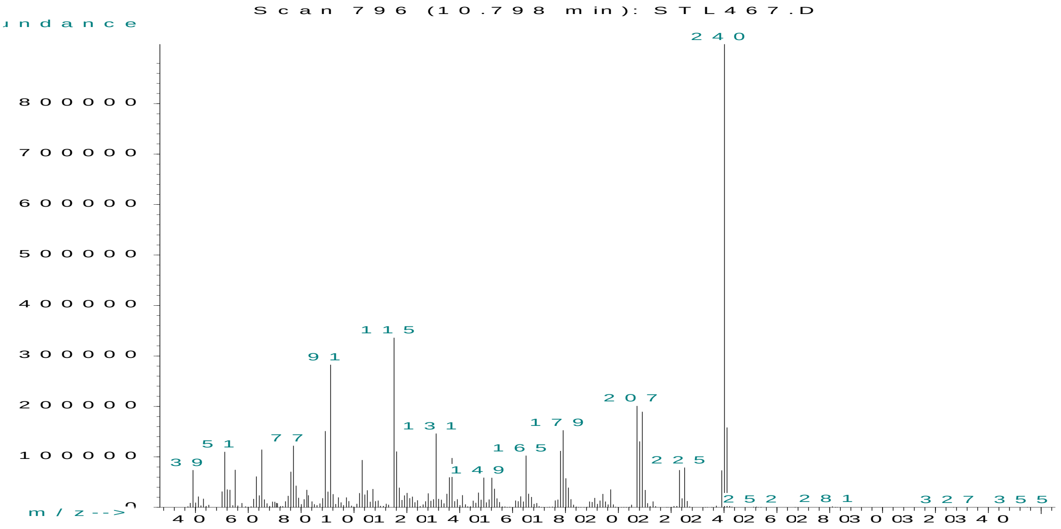

Figure S1. Mass spectrum of (2-methoxy-4-(3-phenyl-2-propen-1-yl)-phenol) (**1E)** and (**1Z**).

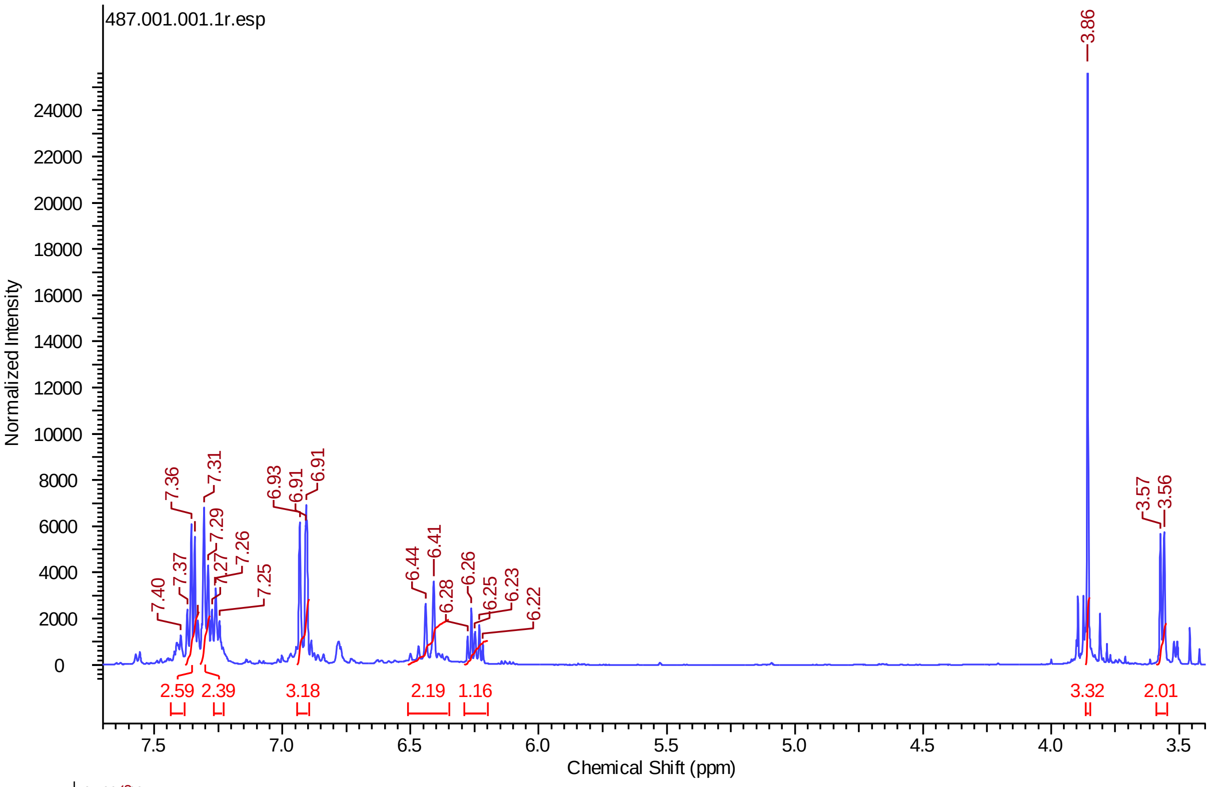
487.001.001.1r.esp
Normalized Intensity
Chemical Shift (ppm)
7.40
7.37
7.36
7.31
7.29
7.27
7.26
7.25
6.93
6.91
6.91
6.44
6.41
6.28
6.26
6.25
6.23
6.22
3.86
3.57
3.56
2.59
2.39
3.18
2.19
1.16
3.32
2.01

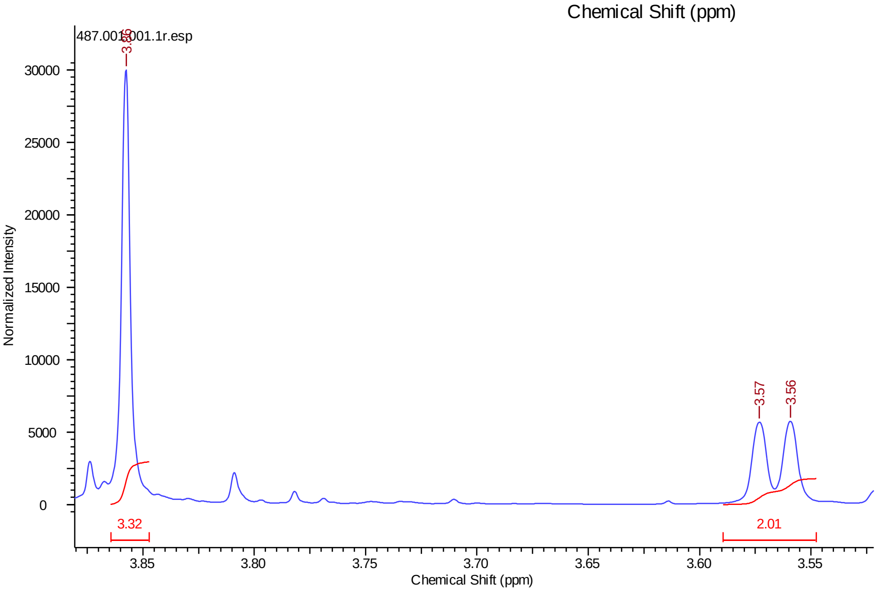
487.001.001.1r.esp
Normalized Intensity
Chemical Shift (ppm)
3.57
3.56
3.32
2.01

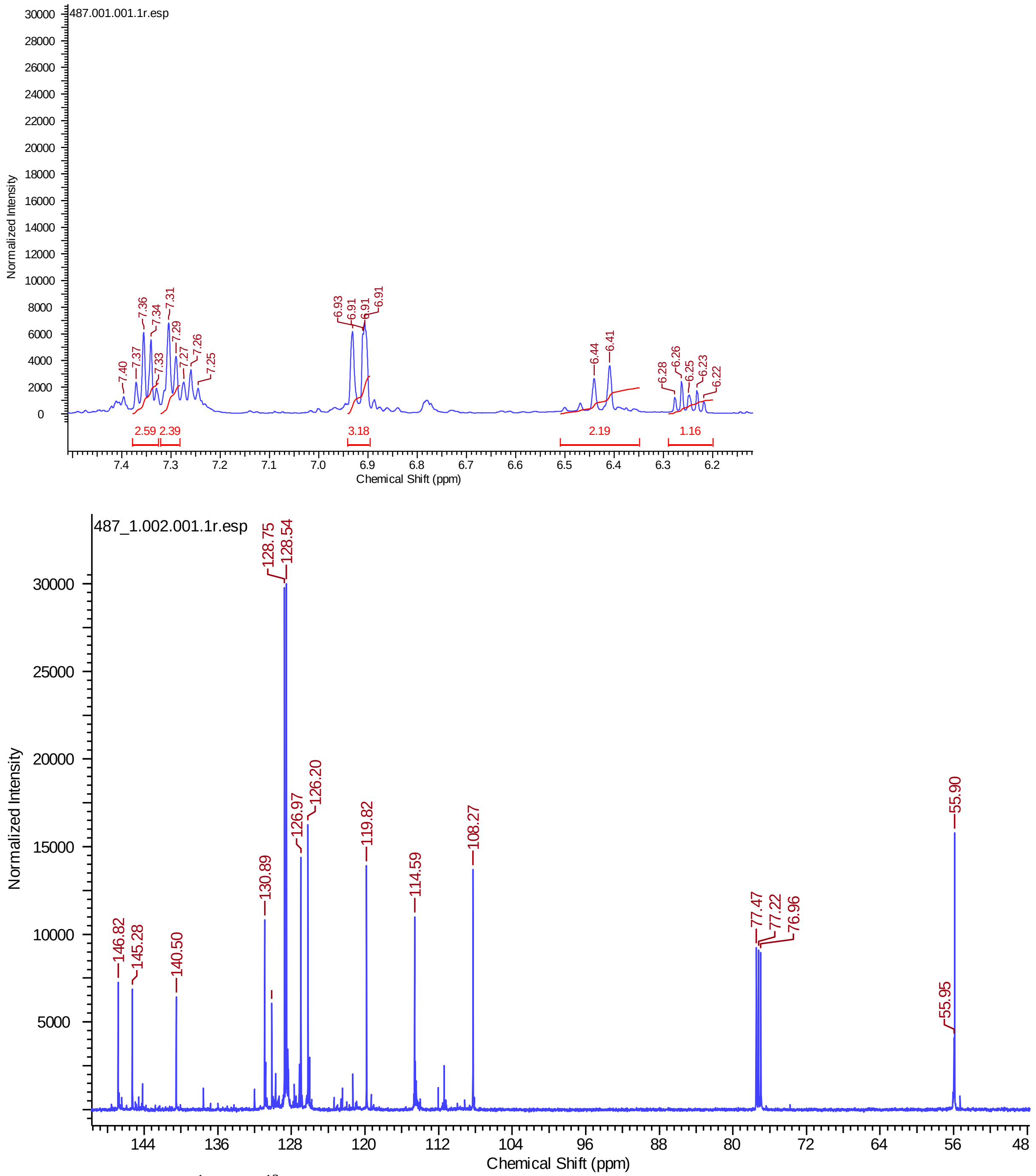

Figure S2. ^{1}H and ^{13}C NMR spectra of (2-methoxy-4-(3-phenyl-2-propen-1-yl)-phenol) (**1E**). Reaction conditions (method A): PhI (1 mmol), eugenol (1 mmol), K_2CO_3 (2 mmol), $Pd(OAc)_2$ (1×10^{-5}mol), DMF:H_2O (4:1), 3h, 100°C

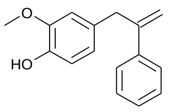

1b (2-methoxy-4-(2-phenylprop-2-en-1-yl)-phenol)
MS: m/z (%) = 77 (24), 103 (24), 137 (100), 225 (17), 240 (85) [M^{+}]

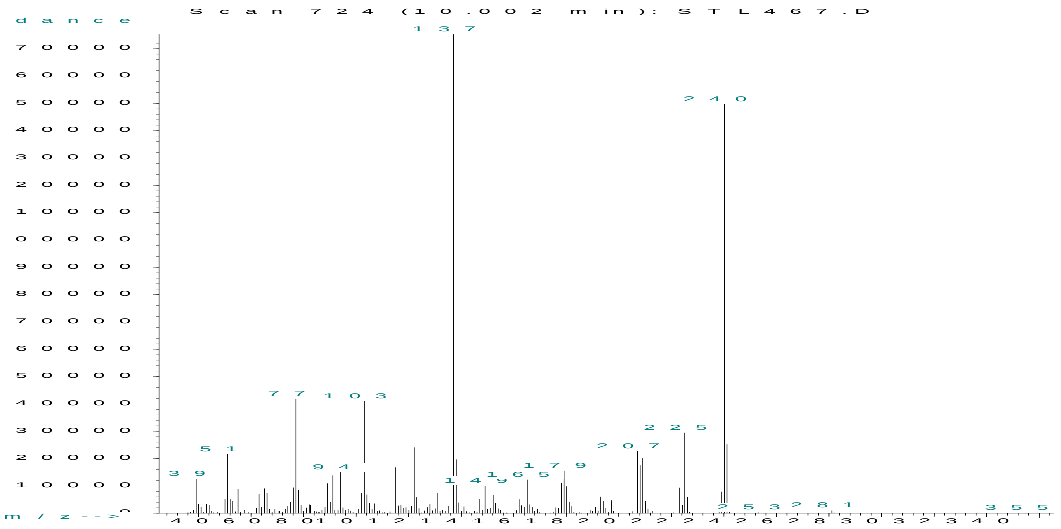

Figure S3. Mass spectrum of 1b (2-methoxy-4-(2-phenylprop-2-en-1-yl)-phenol) (**1b**).

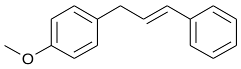

2E/2Z (1-Phenyl-3-(4-methoxyphenyl)-1-propene)

MS: m/z (%) = 77 (21), 91 (25), 115 (61), 121 (31), 165 (14), 178 (14), 193 (27), 209 (21), 224 (100) [M^+] [1]

^{1}H–NMR (500 MHz, $CDCl_3$): δ = 7.49–7.32 (m, 12H, H_{Ar}), 7. 29 (d, J = 8.8, 2H, H_{Ar}), 6.99 (d, 8.8 Hz, 2H, H_{Ar}), 6.96 (d, 8.8 Hz, 2H, H_{Ar}), 6.55 (m, 2H, CH, CH), 6.47 (td, J = 15.6, 6.7 Hz, 1H, CH), 6.35 (td, J = 15.6, 6.7 Hz, 1H, CH), 3.89 (s, 3H, OCH_3), 3.88 (s, 3H, OCH_3), 3.65 (d, J = 6.9, 2H, CH_2), 3.62 (d, J = 6.7, 2H, CH_2) ppm [2].

2E ((E)-1-Phenyl-3-(4-methoxyphenyl)-1-propene)

^{13}C–NMR (125 MHz, $CDCl_3$): δ = 158.29, 137.73, 130.93, 130.65, 130.03, 129.78, 128.67, 127.42, 126.30, 113.95, 55.36, 38.62 ppm [2].

2Z ((Z)- 1-Phenyl-3-(4-methoxyphenyl)-1-propene)

^{13}C–NMR (125 MHz, $CDCl_3$): δ = 159.06, 140.63, 132.32, 130.49, 129.85, 128.84, 128.64, 127.22, 127.20, 114.11, 55.28, 39.51 ppm.

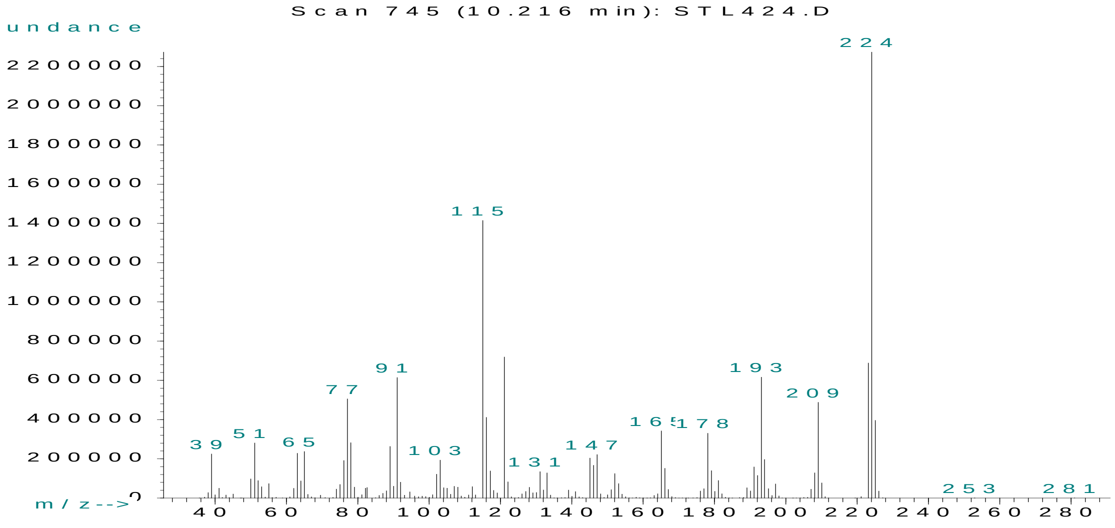

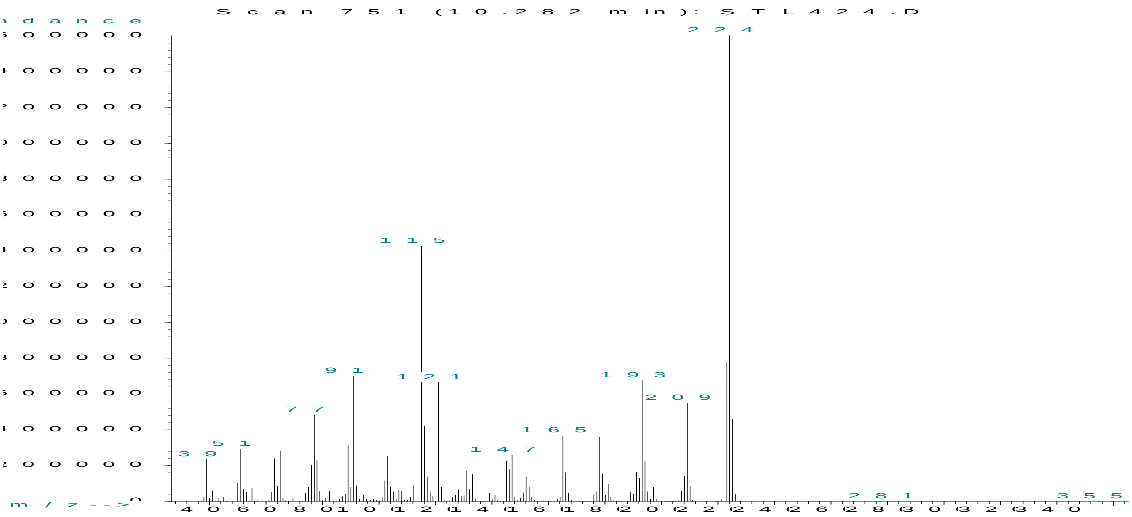

Figure S4. Mass spectrum of 1-Phenyl-3-(4-methoxyphenyl)-1-propene (**2E**) and (**2Z**).

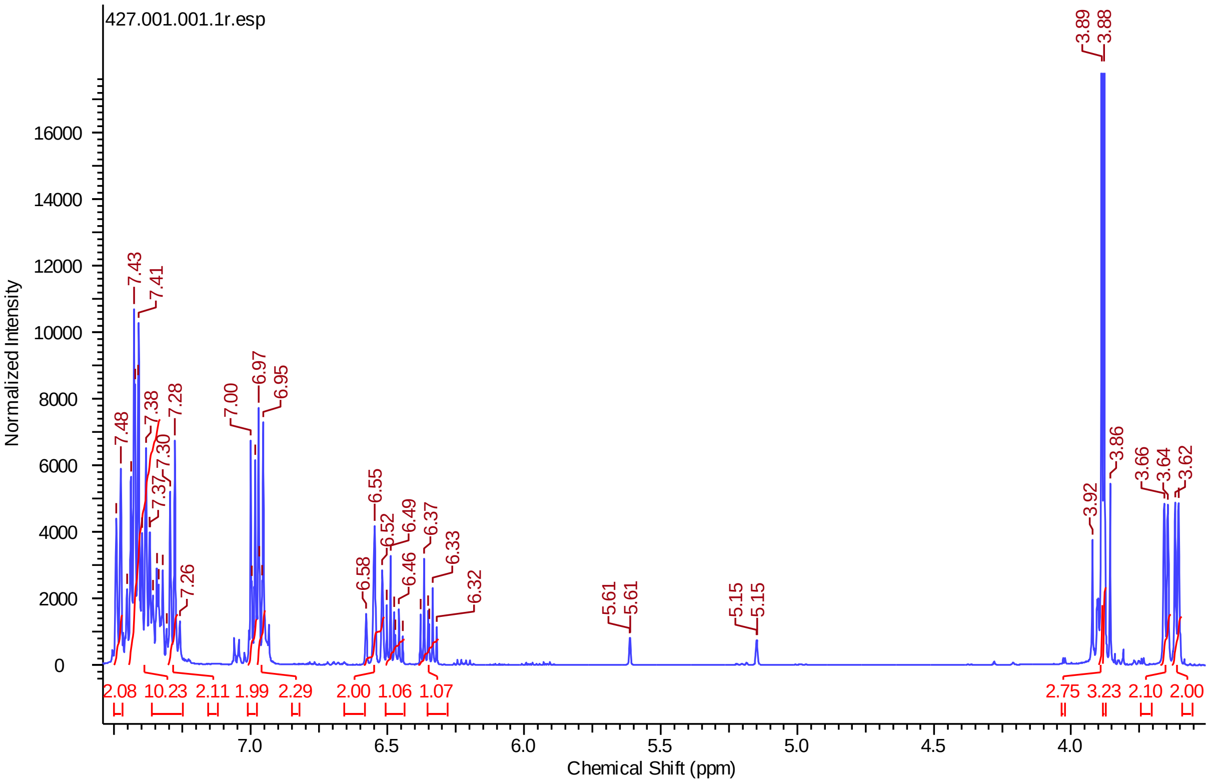

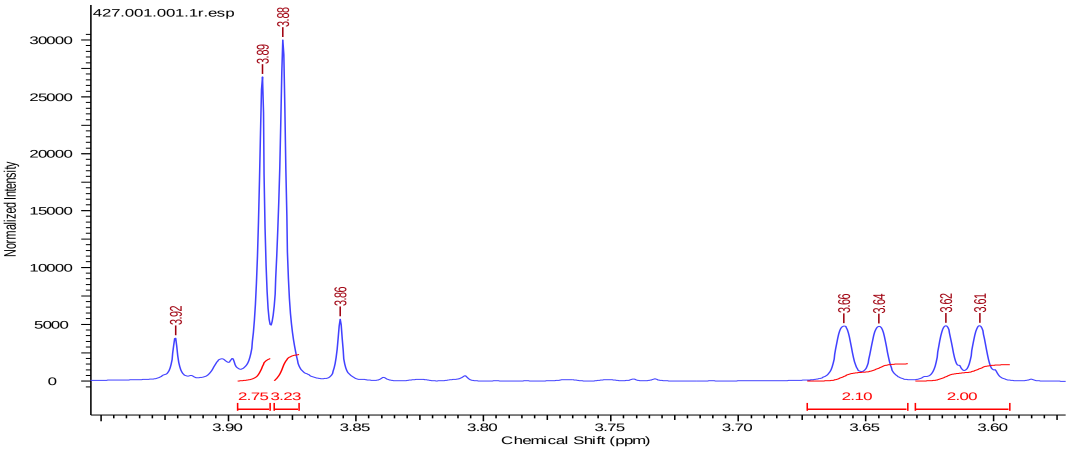

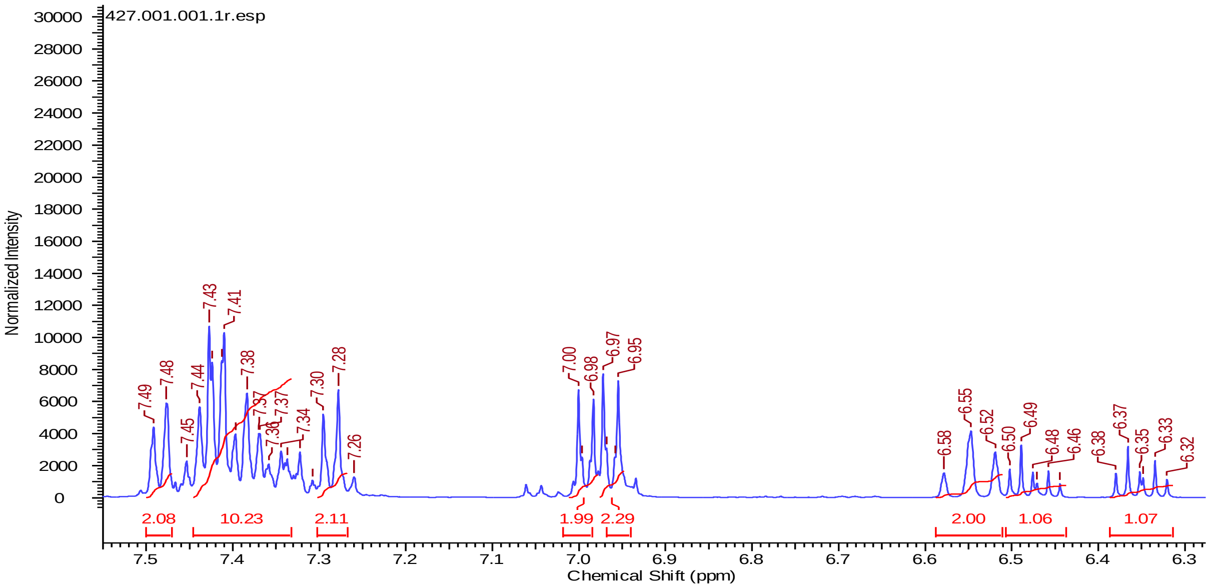

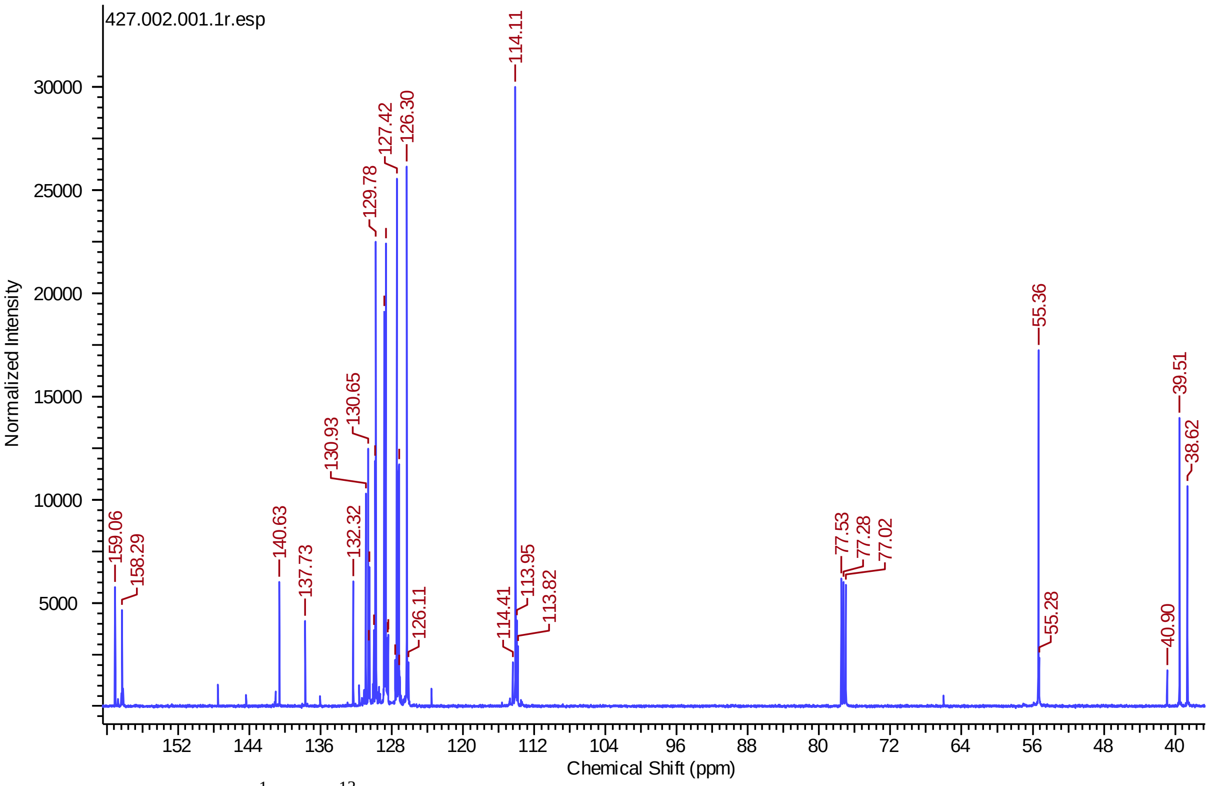

Figure S5. ^{1}H and ^{13}C NMR spectra of 1-Phenyl-3-(4-methoxyphenyl)-1-propene (**2E**).
Reaction conditions (method A): PhI (1 mmol), estragole (1 mmol), K_2CO_3 (2 mmol), $Pd(OAc)_2$ ($1{\times}10^{-5}$mol), DMF (5cm^3), 3h, 100°C

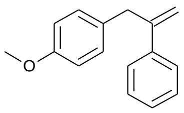

2b (1-methoxy-4-(2-phenyl-2-propen-1-yl)-benzene)
MS: m/z (%) = 77 (23), 103 (13), 121 (100), 193 (10), 209 (10), 224 (45) [M$^+$]

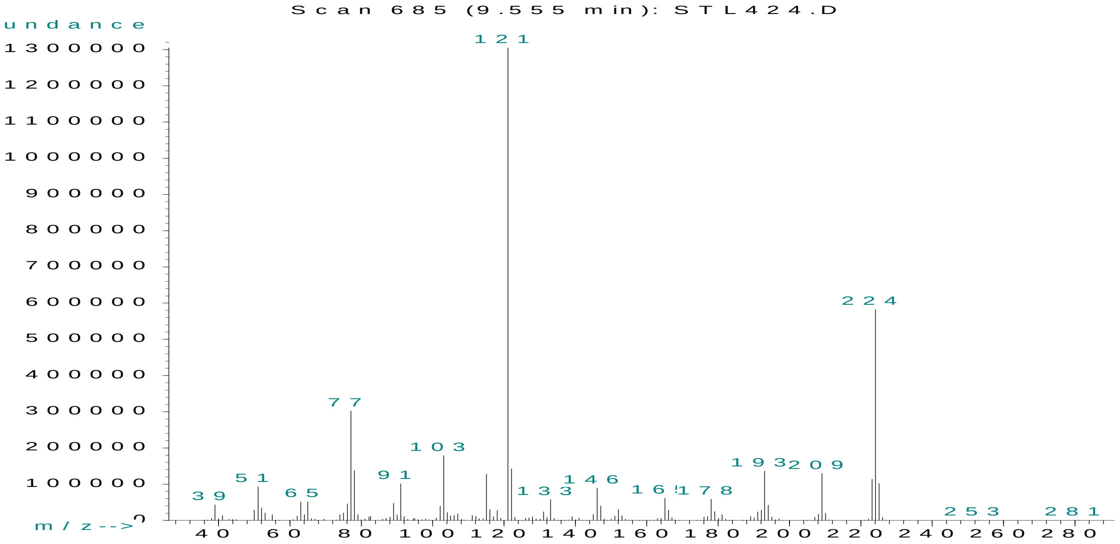

Figure S6. Mass spectrum of (1-methoxy-4-(2-phenyl-2-propen-1-yl)-benzene) (**2b**).

Control experiment 2:

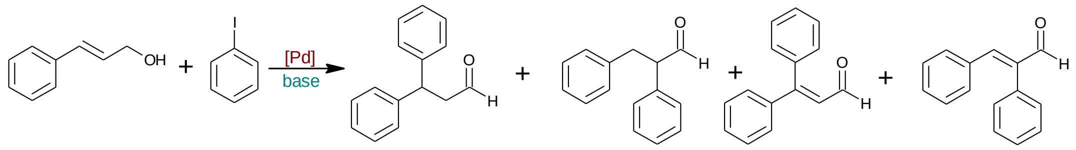

3 4 5 6 (E/Z)

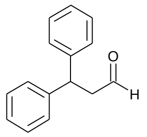

3 (3,3-diphenylpropanal)

MS: m/z (%) = 77 (20), 105 (23), 167 (100), 192 (20), 210 (56) [M^+] [3].

^{1}H–NMR (500 MHz, $CDCl_3$): δ = 9.74 (t, *J* = 1.91, 1H, CHO), 7.25–7.17 (m, 10H, H_{Ar}), 4.63 (t, *J* = 7.82 Hz, 1H, CH), 3.17 (dd, *J* = 7.82, 1.91 Hz, 2H, CH_2) ppm [3,5].

^{13}C–NMR (125 MHz, $CDCl_3$): δ = 198.33, 142.20, 128.26, 127.24, 126.42, 46.95, 42.91 [3].

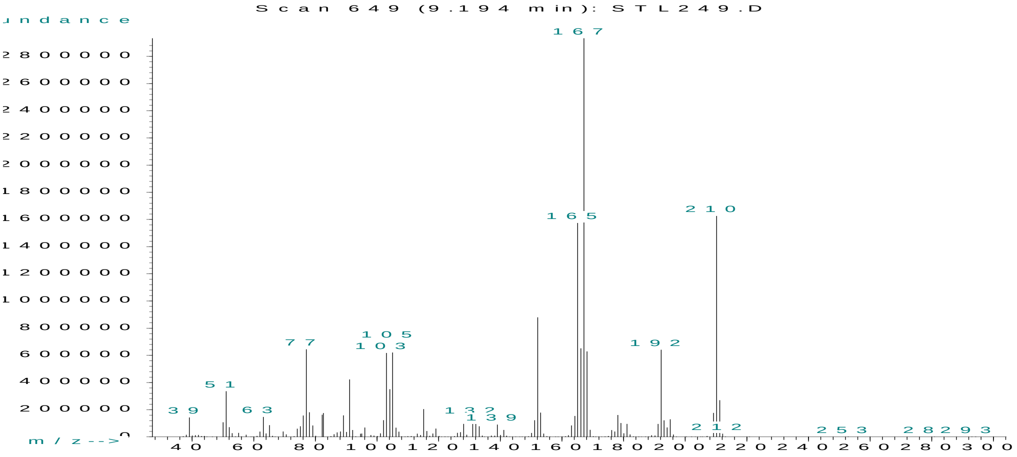

Figure S7. Mass spectrum of 3,3-diphenylpropanal (**3**).

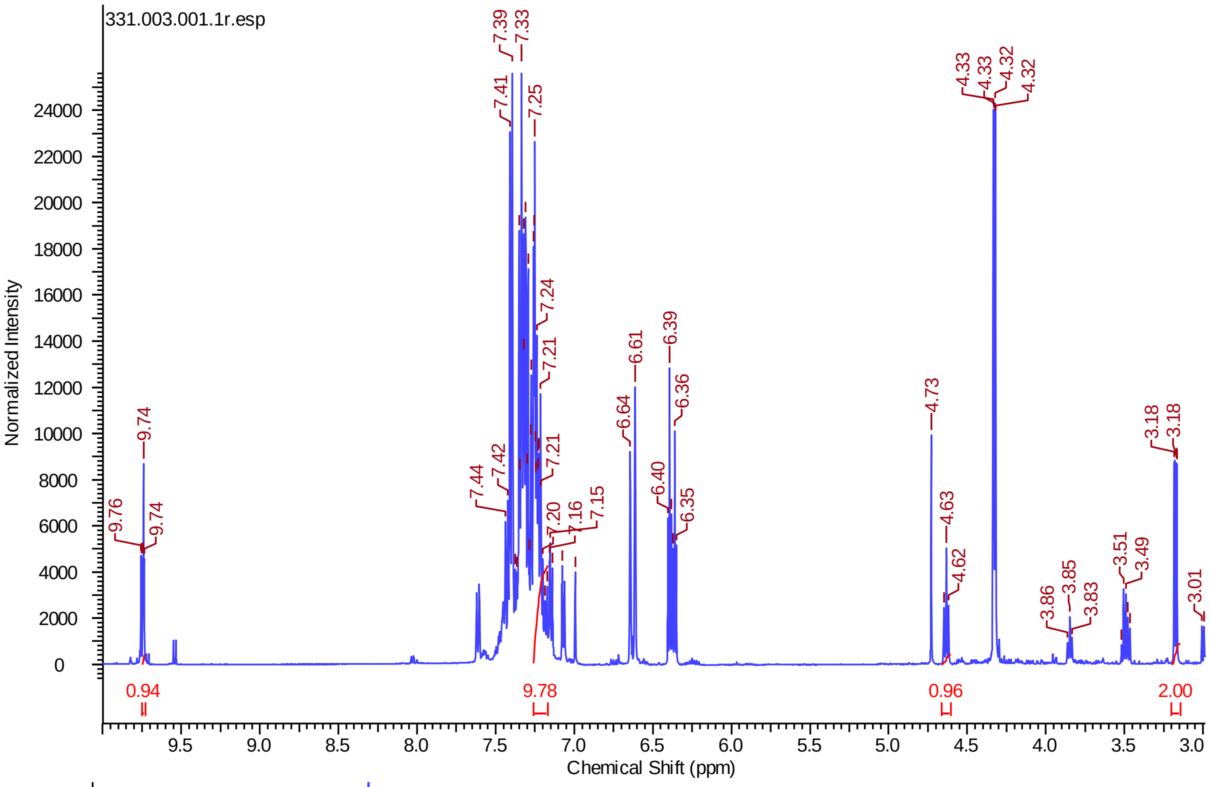

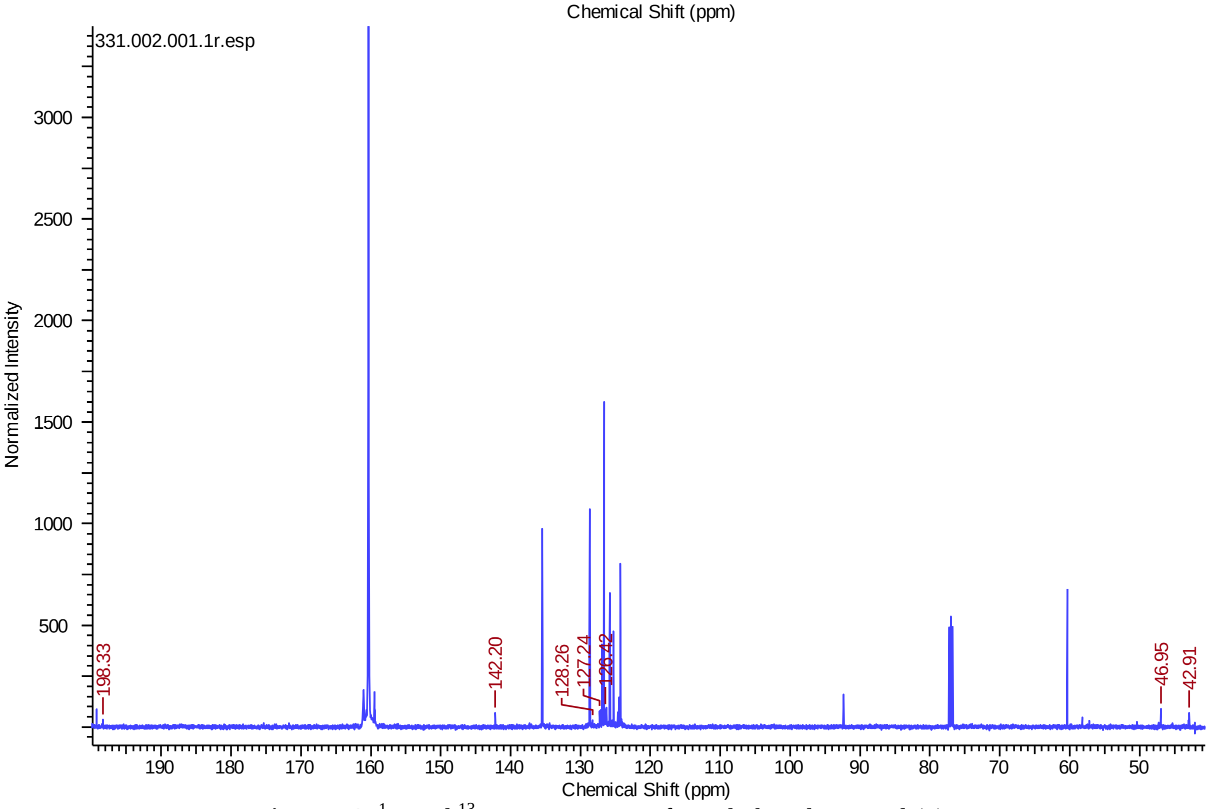

Figure S8. ^{1}H and ^{13}C NMR spectra of 3,3-diphenylpropanal (**3**).
Reaction conditions (method C): PhI (1 mmol), cinnamyl alcohol (1 mmol), K_2HPO_4 (2 mmol), $Pd(OAc)_2$ ($1{\times}10^{-5}$mol), DMF, 6h, 100°C

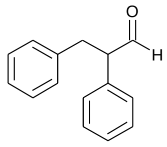

4 (2,3-diphenylpropanal)

MS: m/z (%) = 77 (12), 91 (100), 103 (9), 165 (9), 181 (18), 210 (32) [M^{+}] [4]

^{1}H–NMR (500 MHz, $CDCl_3$): δ = 9.76 (d, J = 1.53, 1H, CHO), 7.31–7.26 (m, 10H, H_{Ar}), 3.85 (t, J = 6.68 Hz, 1H, CH), 3.48 (dd, J = 13.44, 6.68 Hz, 1H, CH_2), 2.98 (dd, J = 14.11, 7.82 Hz, 1H, CH_2) [5] ppm.
^{13}C–NMR (125 MHz, $CDCl_3$): δ = 199.24, 135.47, 128.67, 126.96, 126.63, 125.85, 125.79, 125.26, 124.29, 33.83, 28.68

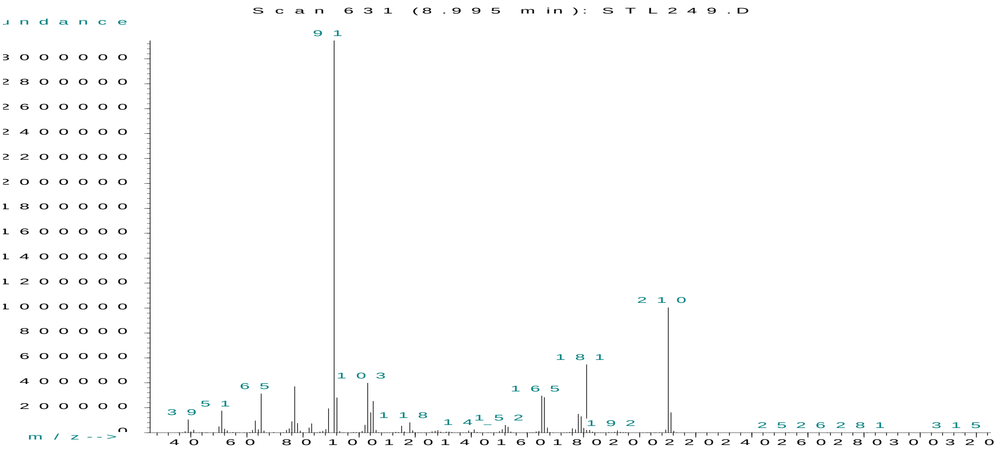

Figure S9. Mass spectrum of 2,3-diphenylpropanal (**4**).

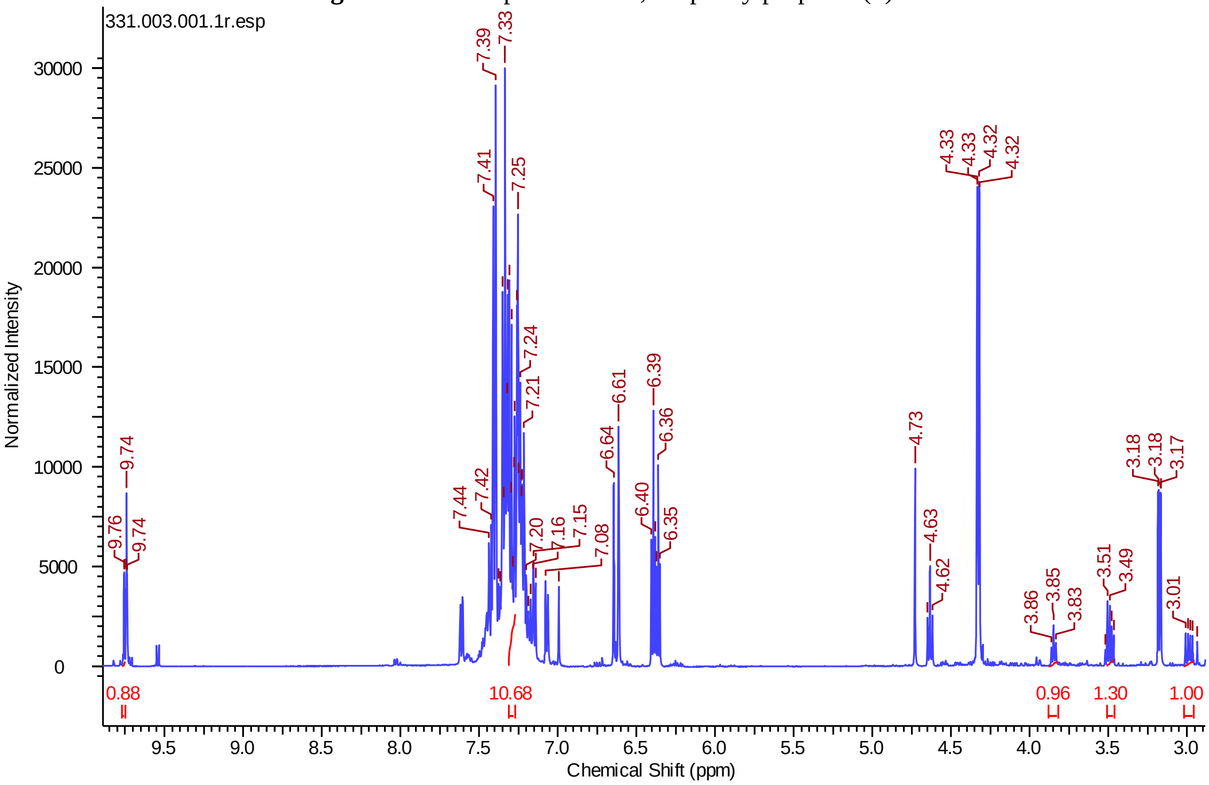

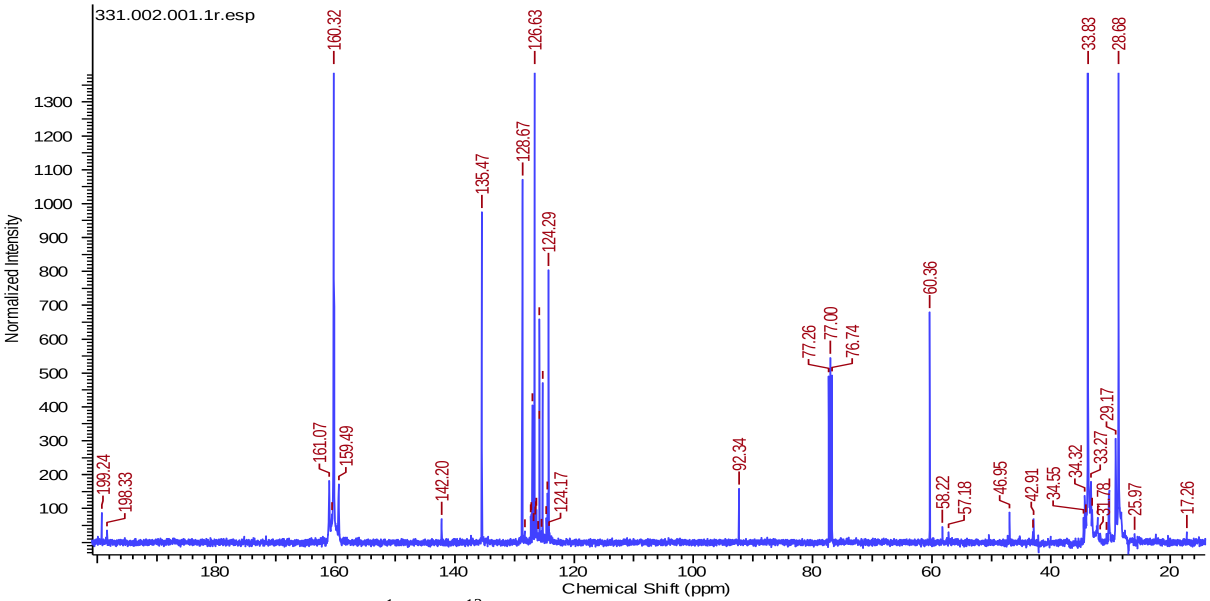

Figure S10. ^{1}H and ^{13}C NMR spectra of 2,3-diphenylpropanal (**4**).

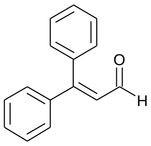

5 (3,3-diphenylprop-2-enal)
MS: m/z (%) = 77 (18), 89 (14), 102 (44), 152 (12), 165 (15), 178 (50), 207 (100), 208 (73) [M$^+$] [6]

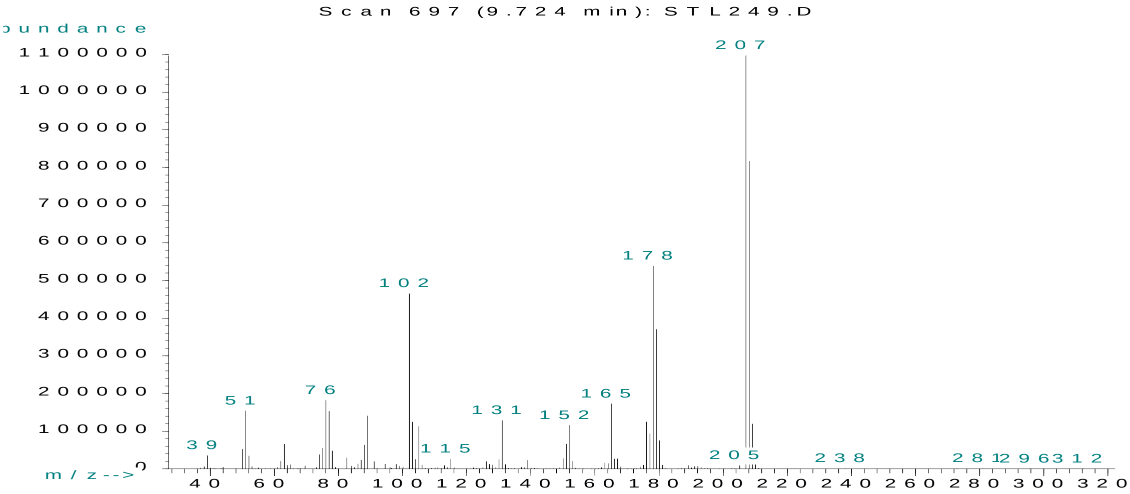

Figure S11. Mass spectrum of 3,3-diphenylprop-2-enal (**5**).

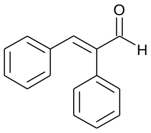

6 ((2*E*)-2,3-diphenylprop-2-enal)
MS: m/z (%) = 77 (18), 89 (18), 102 (44), 152 (20), 165 (26), 178 (79), 207 (56), 208 (100) [M$^+$] [4]

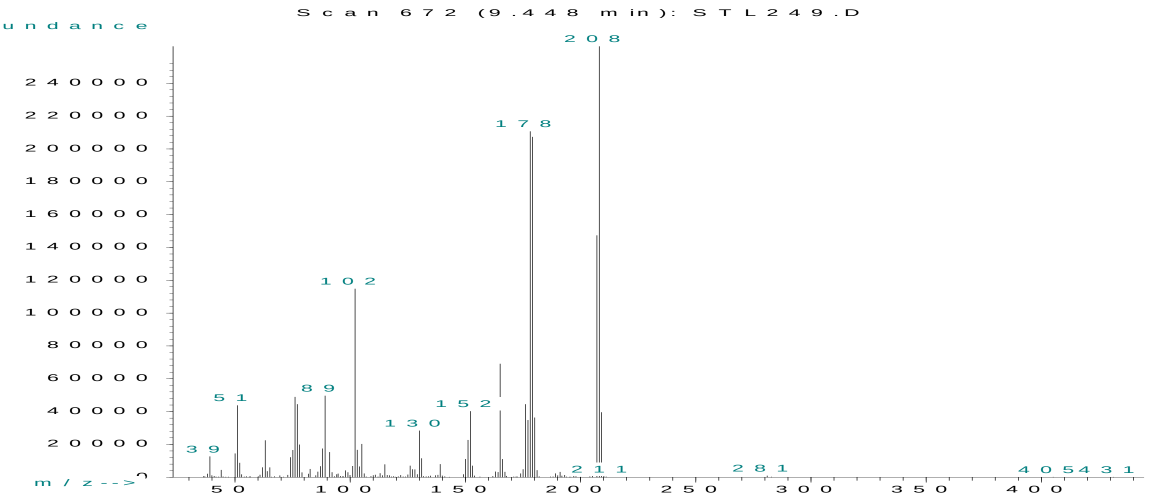

Figure S12. Mass spectrum of (2E)-2,3-diphenylprop-2-enal (**6**).

Control experiment 3:

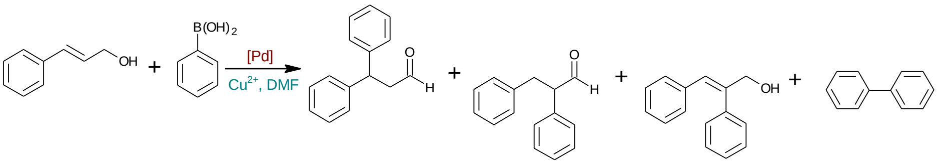

3 4 7

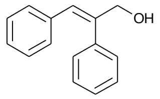

7 (2,3-diphenyl-2-propen-1-ol)

MS: m/z (%) = 77 (18), 91 (54), 105 (100), 165 (114), 178 (25), 210 (46).

^{1}H–NMR (500 MHz, $CDCl_3$): δ = 7.45–7.42 (m, 5H, H_{Ar}), 7.33–7.10 (m, 5H, H_{Ar}), 7.02 (s, 1H, CH), 4.73 (s, 2H, CH_2), 1.86 (s, 1H, OH) ppm.

^{13}C–NMR (125 MHz, $CDCl_3$): δ = 140.59, 138.71, 136.86, 128.89, 128.58, 128.32, 127.62, 127.29, 126.54, 126.23, 68.30 ppm.

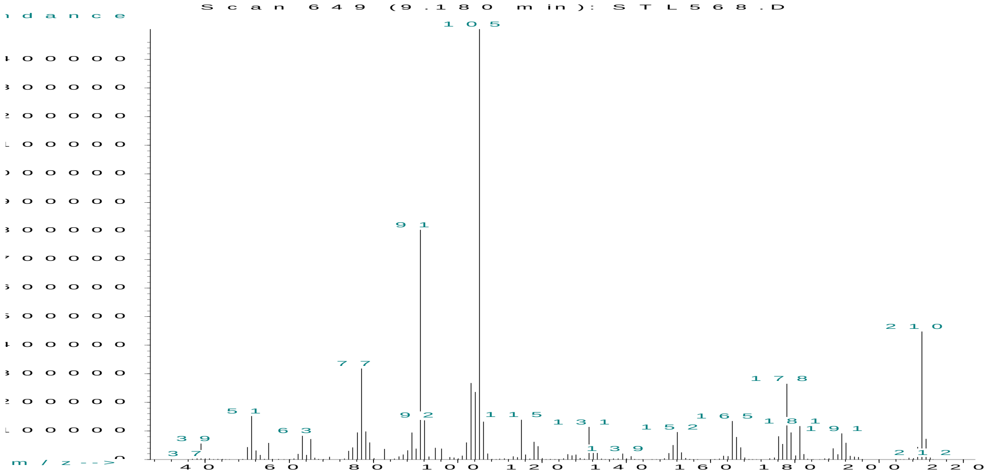

Figure S13. Mass spectrum of 2,3-diphenyl-2-propen-1-ol (**7**).

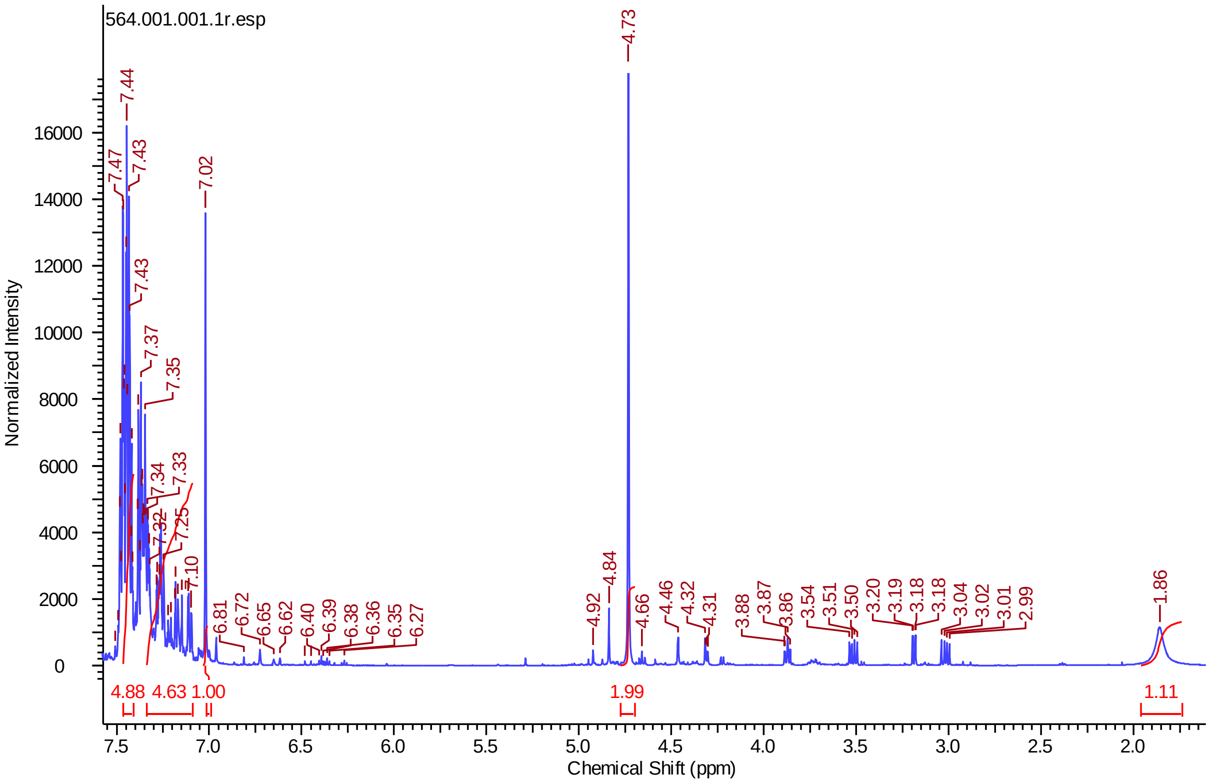

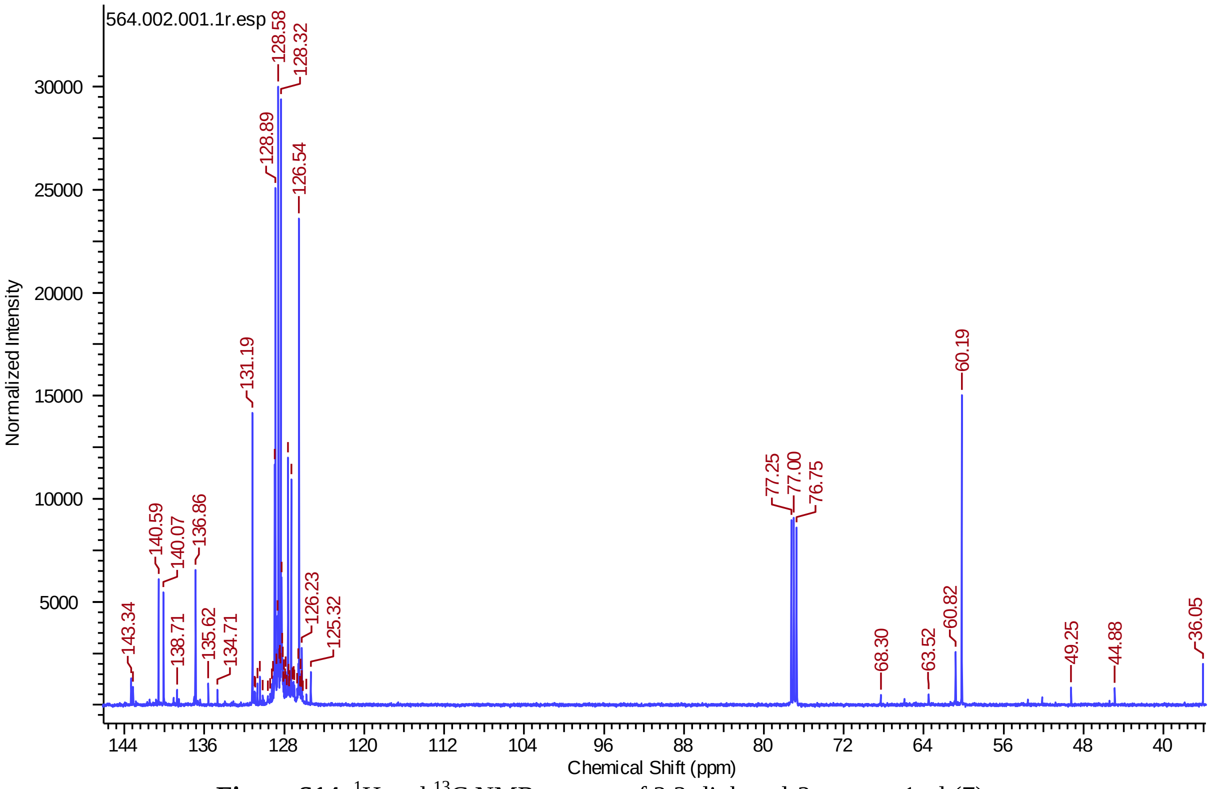

Figure S14. ^{1}H and ^{13}C NMR spectra of 2,3-diphenyl-2-propen-1-ol (**7**).

Control experiment 4:

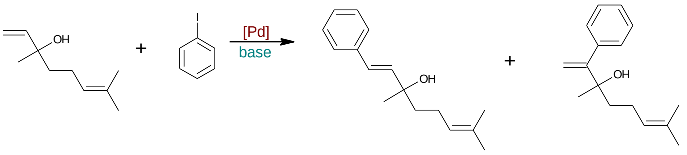

8 9

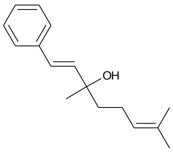

8 ((1*E*)-3,7-dimethyl-1-phenylocta-1,6-dien-3-ol)

MS: m/z (%) = 43 (81), 69 (24), 77 (15), 91 (20), 105 (11), 129 (46), 147 (100), 172 (46), 197 (4), 230 (2) [M^+]

^{1}H–NMR (500 MHz, $CDCl_3$): δ = 7.40 (d, *J* = 7.82 Hz, 2H, H_{Ar}), 7.32 (t, *J* = 7.44 Hz, 2H, H_{Ar}), 7.24 (t, *J* = 7.44 Hz, 1H, H_{Ar}), 6.61 (d, *J* = 16.21 Hz, 1H, CH), 6.29 (d, *J* = 16.02 Hz, 1H, CH), 5.09 (t, *J* = 7.25, 1H, CH), 5.16 (t, *J* = 7.15 Hz, 1H, CH), 2.17–2.03 (m, 2H, CH_2), 1.81 (s, 1H, OH), 1.70 (s, 3H, CH_3), 1.68–1.66 (m, 2H, CH_2), 1.61 (s, 3H, CH_3), 1.40 (s, 3H, CH_3) ppm.

^{13}C–NMR (125 MHz, $CDCl_3$): δ = 137.05, 136.61, 123.02, 128.50, 127.26, 127.09, 126.33, 124.30, 73.43, 42.51, 28.24, 25.66, 22.93, 17.70.

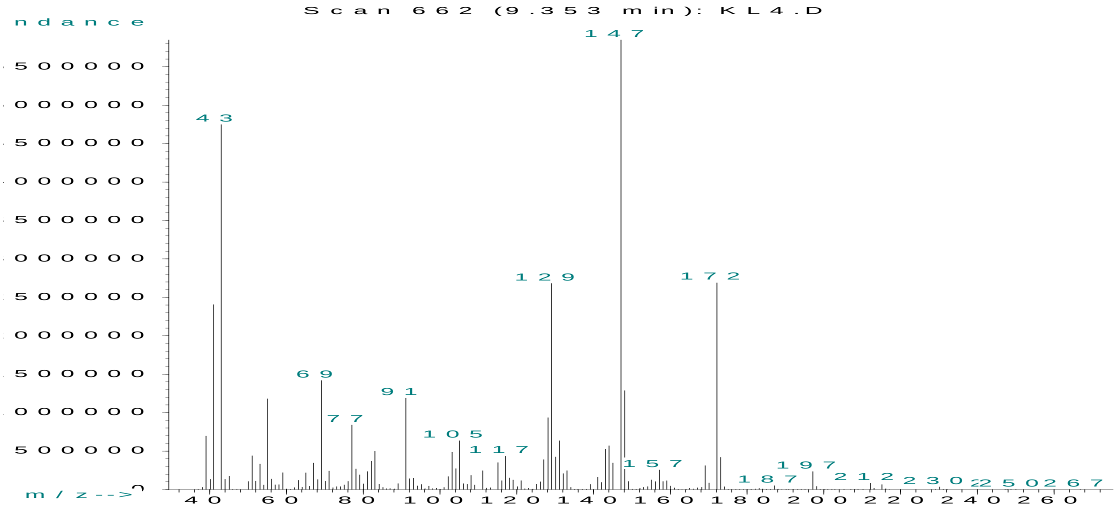

Figure S15. Mass spectrum of (1E)-3,7-dimethyl-1-phenylocta-1,6-dien-3-ol) (**8**).

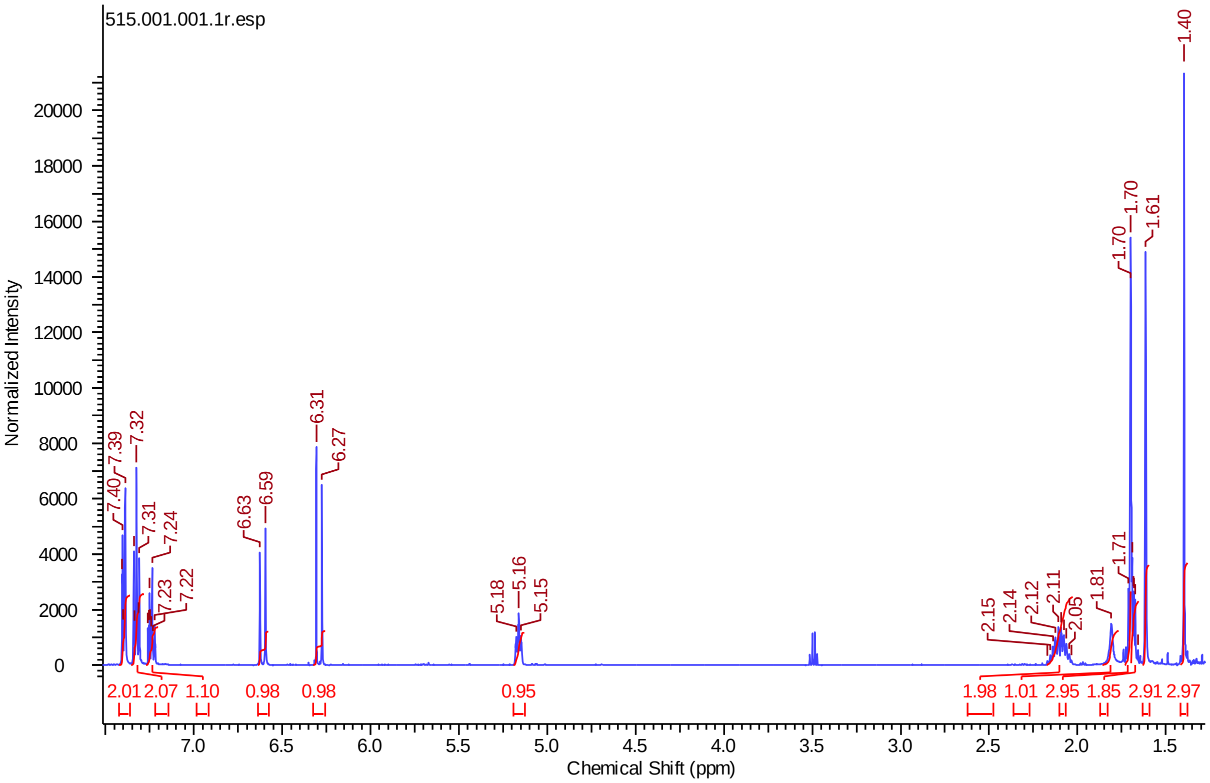

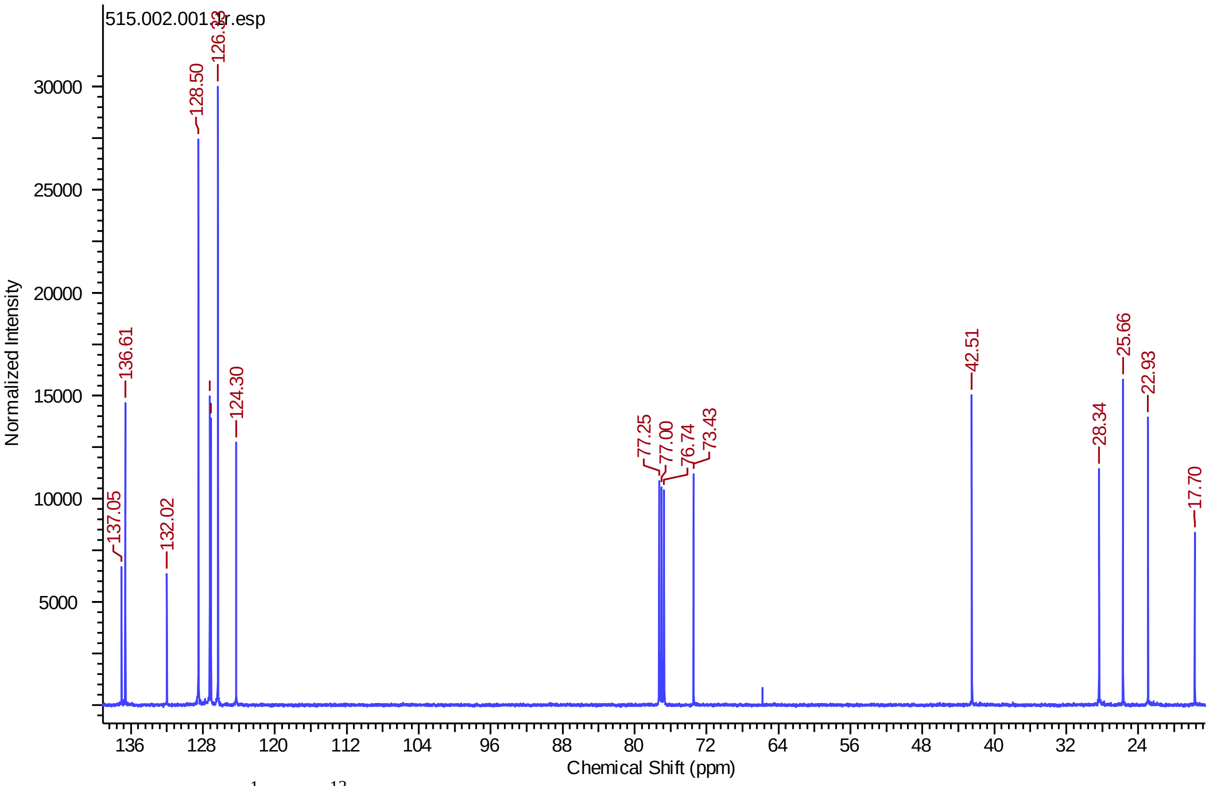

Figure S16. ^{1}H and ^{13}C NMR spectra of (1E)-3,7-dimethyl-1-phenylocta-1,6-dien-3-ol) (**8**). Reaction conditions (method C): PhI (1 mmol), linalool (1 mmol), Et_3N (2 mmol), $Pd(OAc)_2$ (1×10^{-5}mol), DMF, 5h, 100°C

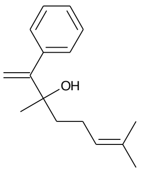

9 (3,7-dimethyl-2-phenylocta-1,6-dien-3-ol)

MS: m/z (%) = 43 (33), 77 (6), 91 (8), 105 (5), 129 (24), 147 (100), 172 (6), 212 (5), 230 (2) [M^+]

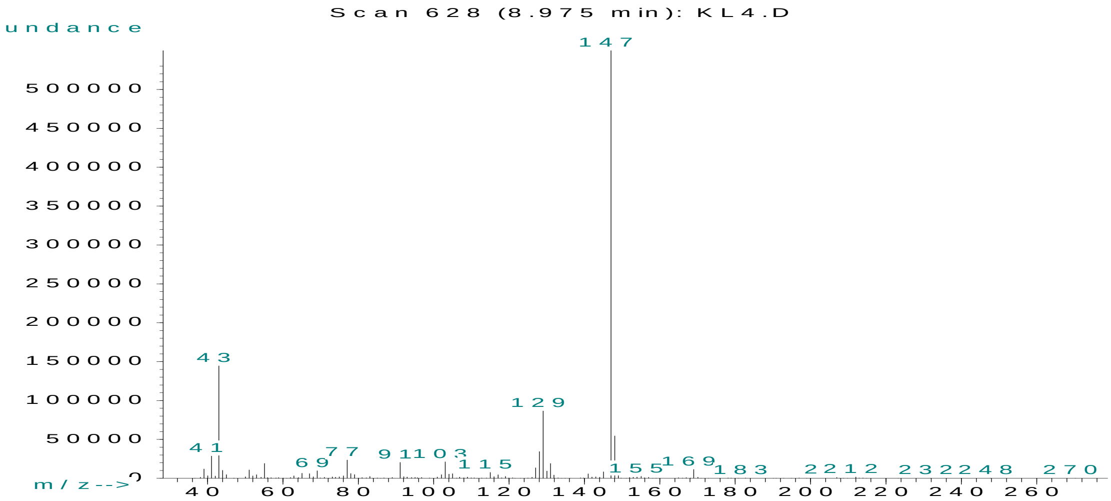

Figure S17. Mass spectrum of (3,7-dimethyl-2-phenylocta-1,6-dien-3-ol) (**9**).

GC examples (preparation of samples for GC was described in the Experimental part)

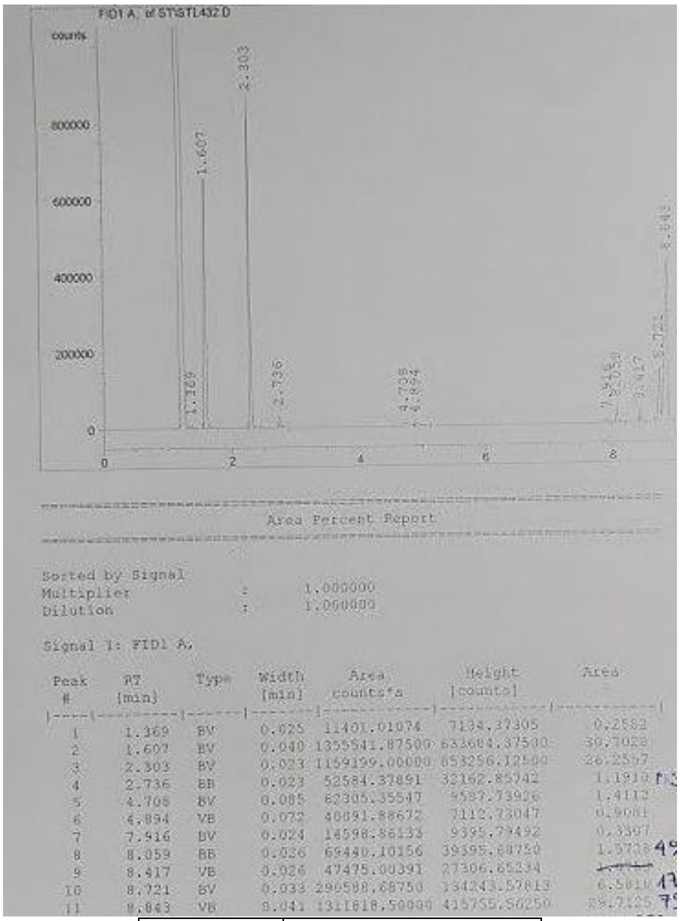

Area Percent Report

Sorted by Signal
Multiplier : 1.000000
Dilution : 1.000000

Signal 1: FID1 A,

Peak #	RT [min]	Type	Width [min]	Area counts*s	Height [counts]	Area
1	1.169	BV	0.025	11401.01074	7134.37305	0.2582
2	1.607	BV	0.040	1355541.87500	633684.37500	30.7028
3	2.303	BV	0.023	1159199.00000	653256.12500	26.2557
4	2.736	BB	0.023	52584.37891	32162.85742	1.1910
5	4.708	BV	0.085	62305.35547	9587.73926	1.4112
6	4.894	VB	0.072	40091.88672	7112.73047	0.9081
7	7.916	BV	0.024	14598.86133	9395.79492	0.3307
8	8.059	BB	0.026	69440.10156	39395.68750	1.5728
9	8.417	VB	0.026	47475.00391	27306.65234	[illegible]
10	8.721	BV	0.033	290588.68750	134243.57813	6.5816
11	8.843	VB	0.041	1311818.50000	415755.56250	29.7125

Peak [min]	Compound
1.607	DMF
2.303	mesitylene
2.736	PhI

4.708	eugenol
4.894	biphenyl
8.059	1b
8.721	1Z
8.843	1E

Figure S18. GC profile for the Heck reaction of PhI with eugenol.
Reaction conditions (method A): PhI (1 mmol), eugenol (1 mmol), K_2CO_3 (2 mmol), $Pd(OAc)_2$ (1×10^{-5}mol), DMF, 1h, 100°C

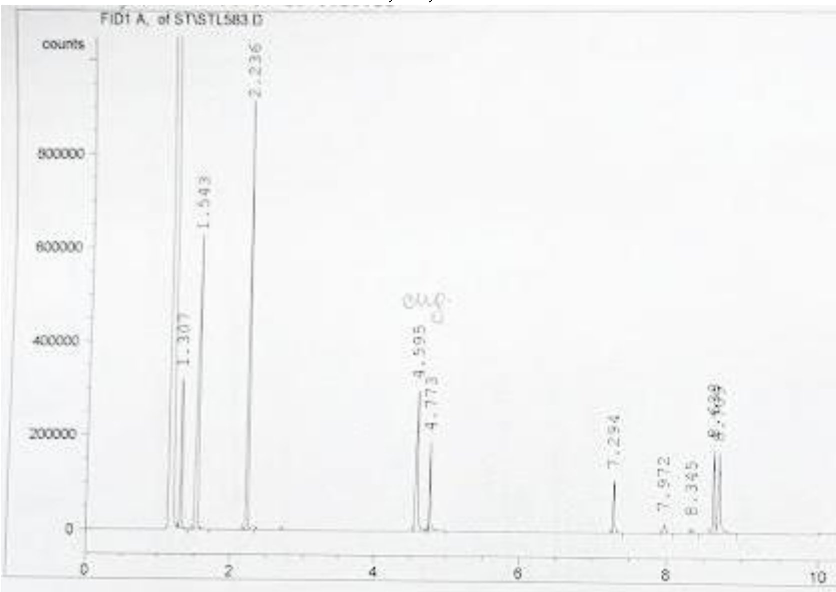

Area Percent Report

Sorted by Signal
Multiplier : 1.000000
Dilution : 1.000000

Signal 1: FID1 A,

Peak #	RT [min]	Type	Width [min]	Area counts*s	Height [counts]	Area %
1	1.307	BB	0.025	483849.56250	314556.09375	9.9130
2	1.543	BV	0.038	1221015.00000	607657.87500	25.0159
3	2.236	BV	0.023	1235882.25000	891354.12500	25.3205
4	4.595	BV	0.031	617736.75000	296262.21875	12.6561
5	4.773	VB	0.020	247421.06250	185548.12500	5.0691
6	7.294	BB	0.022	152039.45313	108085.99219	3.1150
7	7.972	BB	0.032	41403.13672	18864.65625	0.8483
8	8.345	VB	0.040	27751.07812	9486.79395	0.5686
9	8.638	BV	0.024	286688.93750	172208.81250	5.8736
10	8.709	VB	0.030	329643.53125	[illegible]	[illegible]

Peak [min]	Compound
1.543	DMF
2.236	mesitylene
4.595	eugenol
4.773	biphenyl
7.294	1b
8.638	1Z
8.709	1E

Figure S19. GC profile for the Heck-type reaction of eugenol with $PhB(OH)_2$.
Reaction conditions (method B): eugenol (1 mmol), phenylboronic acid (1.5 mmol), Cu^{2+} salt (2 mmol), $Pd(OAc)_2$ (1×10^{-6} mol), DMF (5 cm^3), 6h, 100°C

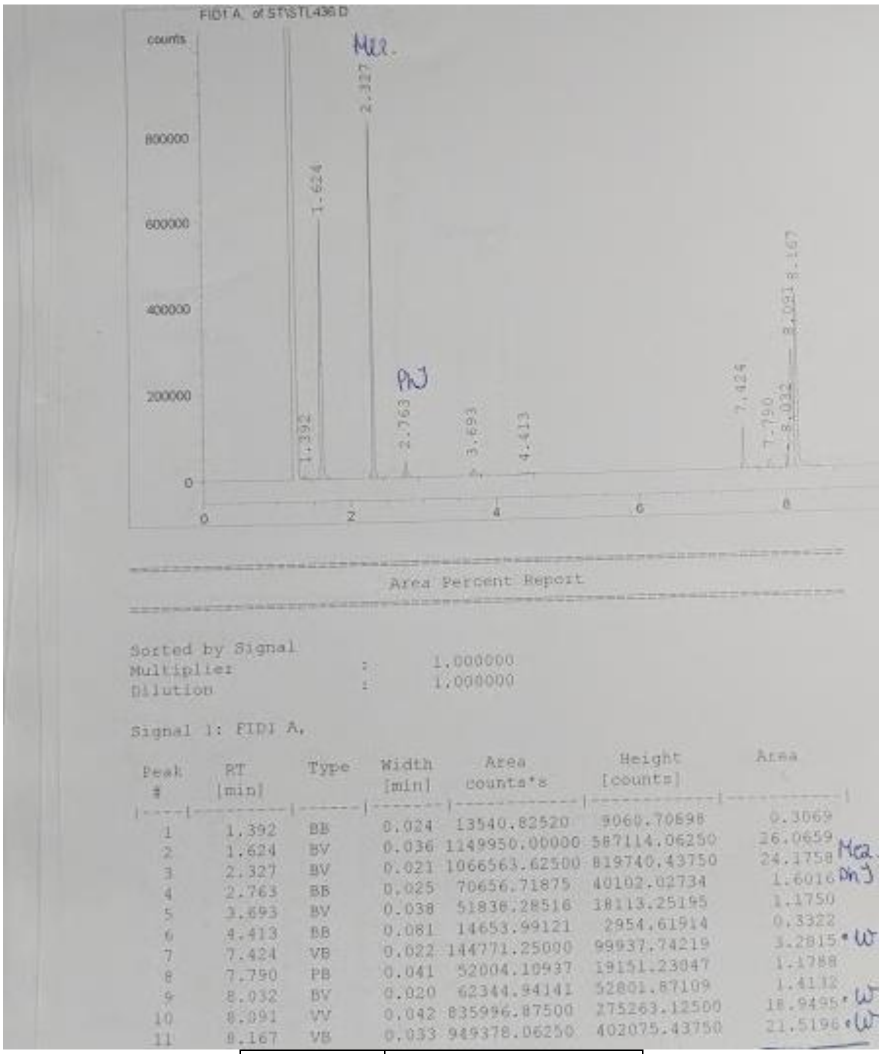

Peak [min]	Coumpound
1.624	DMF
2.327	mesitylene
2.763	PhI
3.693	estragole
7.424	2b
8.091	2Z
8.167	2E

Figure S20. GC profile for the Heck reaction of PhI with estragole.
Reaction conditions (method A): PhI (1 mmol), estragole (1 mmol), K_2CO_3 (2 mmol), $Pd(OAc)_2$ ($1{\times}10^{-5}$mol), DMF (5 cm^3), 3h, 100°C

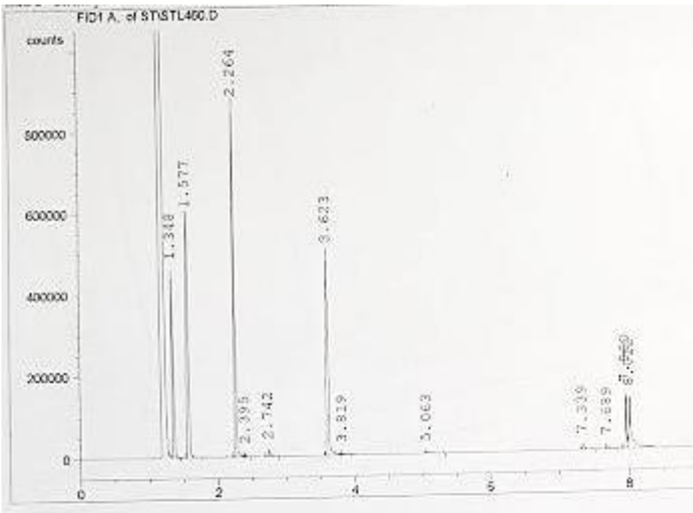

Area Percent Report

Sorted by Signal
Multiplier : 1.000000
Dilution : 1.000000

Signal 1: FID1 A,

Peak #	RT [min]	Type	Width [min]	Area counts*s	Height [counts]	Area %
1	1.348	BB	0.023	638801.62500	456033.06250	14.0785
2	1.577	BV	0.035	1096869.87500	584929.81250	24.1739
3	2.264	BV	0.022	1178769.87500	857309.37500	25.9789
4	2.395	VB	0.024	10662.29199	6377.53076	0.2350
5	2.742	BB	0.048	32129.29883	16528.13477	0.7081
6	3.623	BV	0.028	987972.12500	493426.03125	21.7739
7	3.819	VB	0.041	11152.99512	3537.21289	0.2458
8	5.063	VB	0.105	24272.77539	2932.70386	0.5349
9	7.339	BB	0.032	30413.51172	13167.18066	0.6703
10	7.689	BB	0.040	20561.31445	7031.69580	0.4532
11	7.960	BV	0.022	180715.35937	136361.60938	3.9828
12	8.012	VB	0.036	325092.15625	123457.84687	7.1647

Peak [min]	Compound
1.577	DMF
2.264	mesitylene
3.623	estragole
7.339	2b
7.960	2Z
8.012	2E

Figure S21. GC profile for the Heck-type reaction of estragole with $PhB(OH)_2$.
Reaction conditions (method B): estragole (1 mmol), phenylboronic acid (1.5 mmol), Cu^{2+} salt (2 mmol), $PdCl_2cod$ (1×10^{-5} mol), DMF (5 cm^3), 4h, 100°C

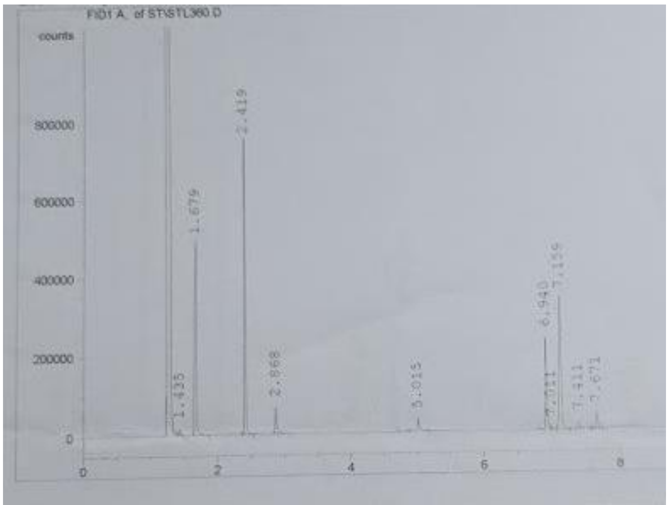

Area Percent Report

Sorted by Signal
Multiplier : 1.000000
Dilution : 1.000000

Signal 1: FID1 A,

Peak #	RT [min]	Type	Width [min]	Area counts*s	Height [counts]	Area %
1	1.435	BB	0.021	21254.88281	15744.25879	0.6250
2	1.679	BV	0.034	924740.87500	483959.75000	27.1906
3	2.419	BV	0.023	1015545.25000	740161.18750	29.8606
4	2.868	BB	0.025	117448.63281	68396.36719	3.4534
5	5.015	BB	0.033	73668.81250	30533.07617	2.1661
6	6.940	BV	0.027	406321.56250	230231.34375	11.9473
7	7.011	VV	0.035	11218.70410	4523.31299	0.3299
8	7.159	VB	0.032	736217.50000	326494.81250	21.6474
9	7.411	BB	0.029	39606.75781	19234.05859	1.1646
10	7.671	BB	0.023	54930.76563	37225.14844	1.6152

**Peak [min]**	**Compound**
1.679	DMF
2.419	mesitylene
2.868	PhI
5.015	biphenyl
6.940	3
7.159	4
7.411	6
7.671	5

Figure S22. GC profile for the Heck reaction of cinnamyl alcohol with PhI.
Reaction conditions (method C): PhI (1 mmol), cinnamyl alcohol (1 mmol), NaOAc (2 mmol), $PdCl_2cod$ (1×10^{-5} mol), DMF (5 cm^3), TBAB (1g), 6h, 100 °C

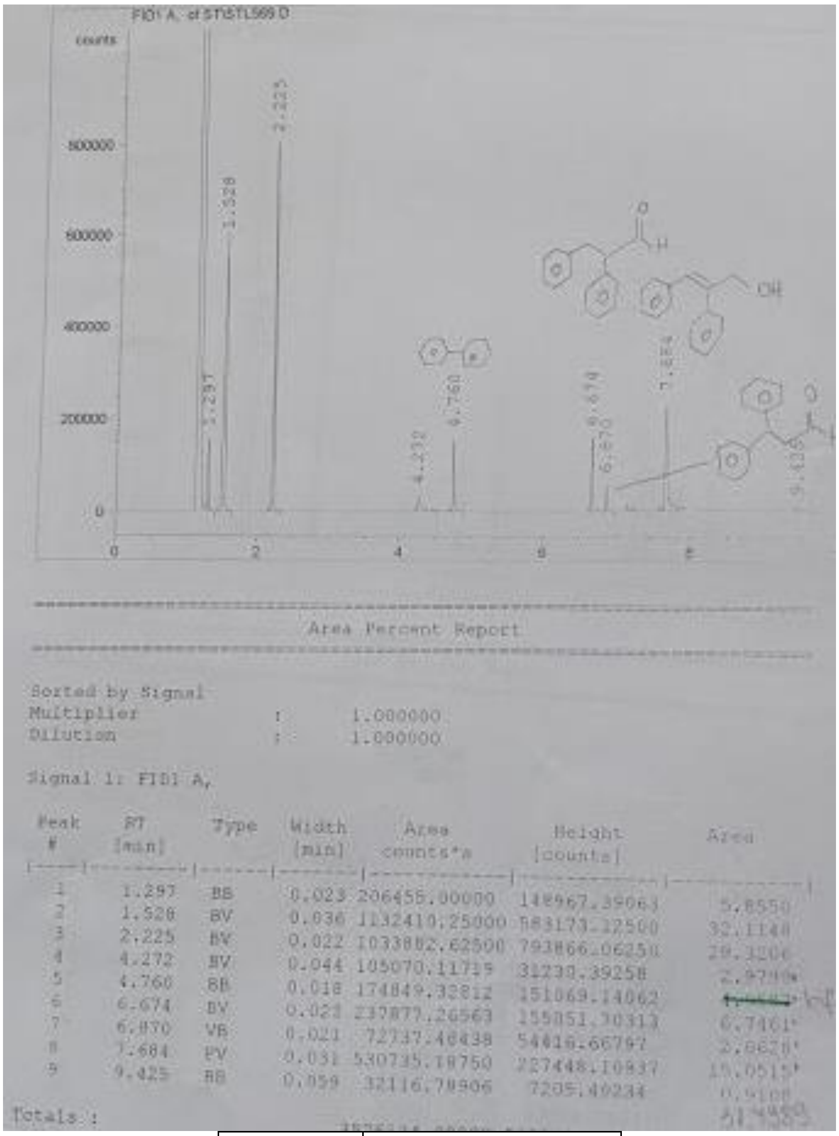

Peak [min]	Compound
1.528	DMF
2.225	mesitylene
4.272	cinnamyl alcohol
4.760	biphenyl
6.674	4
6.870	3
7.784	7

Figure S23. GC profile for the Heck-type reaction of cinnamyl alcohol with $PhB(OH)_2$.
Reaction conditions (method B): cinnamyl alcohol (1 mmol), phenylboronic acid (1.5 mmol), Cu^{2+} salt (2 mmol), Pd_2dba_3 (1×10^{-5} mol), DMF (5 cm^3), 0.5h, 50^oC

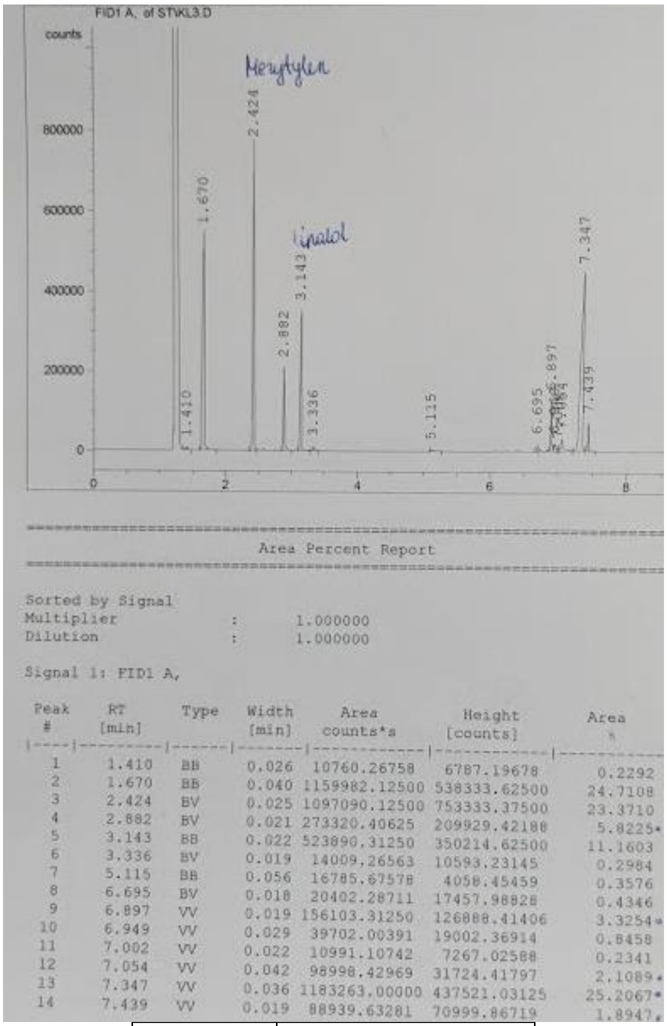

Area Percent Report

Sorted by Signal
Multiplier : 1.000000
Dilution : 1.000000

Signal 1: FID1 A,

Peak #	RT [min]	Type	Width [min]	Area counts*s	Height [counts]	Area %
1	1.410	BB	0.026	10760.26758	6787.19678	0.2292
2	1.670	BB	0.040	1159982.12500	538333.62500	24.7108
3	2.424	BV	0.025	1097090.12500	753333.37500	23.3710
4	2.882	BV	0.021	273320.40625	209929.42188	5.8225*
5	3.143	BB	0.022	523890.31250	350214.62500	11.1603
6	3.336	BV	0.019	14009.26563	10593.23145	0.2984
7	5.115	BB	0.056	16785.67578	4058.45459	0.3576
8	6.695	BV	0.018	20402.28711	17457.98828	0.4346
9	6.897	VV	0.019	156103.31250	126888.41406	3.3254*
10	6.949	VV	0.029	39702.00391	19002.36914	0.8458
11	7.002	VV	0.022	10991.10742	7267.02588	0.2341
12	7.054	VV	0.042	98998.42969	31724.41797	2.1089*
13	7.347	VV	0.036	1183263.00000	437521.03125	25.2067*
14	7.439	VV	0.019	88939.63281	70999.86719	1.8947*

Peak [min]	Compound
1.670	DMF
2.424	mesitylene
3.143	linalool
3.336	linalool
5.115	biphenyl
6.897	9
7.054	8
7.347	8

Figure S24. GC profile for the Heck reaction of linalool with PhI.
Reaction conditions (method C): PhI (1 mmol), linalool (1 mmol), NaOAc (2 mmol), $PdCl_2$cod (1×10^{-5} mol), DMF (5 cm^3), 3h, 140°C

References

1. Wang, J.; Huang, W.; Zhang, Z.; Xiang, X.; Liu, R.; Zhou X. $FeCl_3\cdot6H_2O$ Catalyzed Disproportionation of Allylic Alcohols and Selective Allylic Reduction of Allylic Alcohols and Their Derivatives with Benzyl Alcohol. *J. Org. Chem.* **2009**, 74, 3299–3304, DOI: 10.1021/jo900070q
2. Ortar, G. Palladium-catalyzed cross-coupling reaction of allyl acetates with pinacol aryl- and vinylboronates. *Tetrahedron Lett.* **2003**, 44, 4311–4314, DOI: 10.1016/S0040-4039(03)00980-8
3. Lerebours, R.; Wolf, C. Palladium(II)-Catalyzed Conjugate Addition of Arylsiloxanes in Water. *Org. Lett.* **2007**, 9(14), 2737–2740, DOI: 10.1021/ol071067v

4. Calo, V.; Nacci, A.; Monopoli, A.; Cotugno, P. Palladium-Nanoparticle-Catalysed Ullmann Reactions in Ionic Liquids with Aldehydes as the Reductants: Scope and Mechanism. *Chem. Eur. J.* **2009**, 15, 1272–1279, DOI: 10.1002/chem.200801621
5. Calo, V.; Nacci, A,; Monopoli, A.; Ferola, V. Palladium-Catalyzed Heck Arylations of Allyl Alcohols in Ionic Liquids: Remarkable Base Effect on the Selectivity. *J. Org. Chem.* **2007**, 72, 2596–2601, DOI: 10.1021/jo070005f
6. Cadierno, V.; Garcia-Garrido, S. E.; Gimeno, J. Isomerization of Propargylic Alcohols into a,b-Unsaturated Carbonyl Compounds Catalyzed by the Sixteen-Electron Allyl-Ruthenium(II) Complex [Ru(h^3-2-C_3H_4Me)(CO)(dppf)][SbF_6]. *Adv. Synth. Catal.* **2006**, 348, 101–110, DOI: 10.1002/adsc.200505294
